# Mitochondrial pyruvate supports lymphoma proliferation by fueling a glutamate pyruvate transaminase 2–dependent glutaminolysis pathway

**DOI:** 10.1126/sciadv.abq0117

**Published:** 2022-09-30

**Authors:** Peng Wei, Alex J. Bott, Ahmad A. Cluntun, Jeffrey T. Morgan, Corey N. Cunningham, John C. Schell, Yeyun Ouyang, Scott B. Ficarro, Jarrod A. Marto, Nika N. Danial, Ralph J. DeBerardinis, Jared Rutter

**Affiliations:** ^1^ Department of Biochemistry, University of Utah School of Medicine, Salt Lake City, UT 84112, USA.; ^2^ Department of Cancer Biology, Dana-Farber Cancer Institute, Harvard Medical School, Boston, MA 02115, USA.; ^3^ Blais Proteomics Center, Dana-Farber Cancer Institute, Harvard Medical School, Boston, MA 02215, USA.; ^4^ Department of Cell Biology, Harvard Medical School, Boston, MA 02115, USA.; ^5^ Children’s Medical Center Research Institute, University of Texas (UT) Southwestern Medical Center, Dallas, TX 75390, USA.; ^6^ Howard Hughes Medical Institute, UT Southwestern Medical Center, Dallas, TX 75390, USA.; ^7^ Howard Hughes Medical Institute, University of Utah School of Medicine, Salt Lake City, UT 84112, USA.

## Abstract

The fate of pyruvate is a defining feature in many cell types. One major fate is mitochondrial entry via the mitochondrial pyruvate carrier (MPC). We found that diffuse large B cell lymphomas (DLBCLs) consume mitochondrial pyruvate via glutamate-pyruvate transaminase 2 to enable α-ketoglutarate production as part of glutaminolysis. This led us to discover that glutamine exceeds pyruvate as a carbon source for the tricarboxylic acid cycle in DLBCLs. As a result, MPC inhibition led to decreased glutaminolysis in DLBCLs, opposite to previous observations in other cell types. We also found that MPC inhibition or genetic depletion decreased DLBCL proliferation in an extracellular matrix (ECM)–like environment and xenografts, but not in a suspension environment. Moreover, the metabolic profile of DLBCL cells in ECM is markedly different from cells in a suspension environment. Thus, we conclude that the synergistic consumption and assimilation of glutamine and pyruvate enables DLBCL proliferation in an extracellular environment-dependent manner.

## Teaser

3D culture reveals how the mitochondrial pyruvate carrier supports proliferation in lymphoma.

## INTRODUCTION

The central pathway of carbohydrate metabolism is the conversion of glucose to pyruvate via glycolysis in the cytosol. The subsequent fate of this pyruvate is a critical metabolic node in mammalian cells. In most differentiated cells, the mitochondrial pyruvate carrier (MPC) transports pyruvate into mitochondria where it is used to fuel oxidation and anabolic reactions (*1*). In contrast, in stem cells and many cancers, pyruvate is primarily converted to lactate and excreted from the cell (*2*), a process known as the Warburg effect. Several groups have shown in a variety of tumor types that the Warburg effect can be caused by low activity of the MPC (*3*–*6*). Moreover, re-expression of the MPC in colon cancer cells, which have very low native expression, not only increased mitochondrial pyruvate oxidation but also repressed tumor growth (*3*). However, repression of the MPC is not a universal feature of all cancers. In prostate cancer, high MPC activity is required for lipogenesis and oxidative phosphorylation, and in hepatocellular carcinoma, high MPC activity is required to supply mitochondrial pyruvate for the tricarboxylic acid (TCA) cycle (*7*, *8*). Therefore, we lack a unified understanding of the relationship between a given cancer type and its dependence on the MPC and mitochondrial pyruvate.

Diffuse large B cell lymphomas (DLBCLs) are the most common type of non-Hodgkin lymphoma and are genetically and phenotypically heterogeneous (*9*, *10*). This genetic heterogeneity has been captured by independent classification schemes (*11*, *12*). In particular, consensus cluster classification has identified three subgroups of DLBCL based on gene expression and metabolic signatures: B cell receptor (BCR)–DLBCLs, which are characterized by the expression of genes encoding BCR signaling pathways; OxPhos-DLBCLs, which have high expression of genes involved in mitochondrial oxidative phosphorylation; and HR-DLBCLs, which have increased expression of genes involved in host inflammatory infiltration (*12*–*14*).

In terms of metabolism, OxPhos-DLBCLs display greater fatty acid oxidation than BCR-DLBCLs, whereas BCR-DLBCLs have a higher rate of glycolysis (*13*). However, the qualitative and quantitative differences of carbohydrate metabolism and the broader spectrum of metabolic substrates feeding the TCA cycle, between Oxphos-DLBCLs and BCR-DLBCLs, including glutaminolysis and the interplay between different fuels, are not fully understood. Understanding these basic features of metabolism in these two DLBCL subgroups might inform their therapeutic vulnerabilities.

Although DLBCLs primarily form solid tumors (*15*, *16*), they are routinely passaged and studied in suspension media in the laboratory. Mimicry of the native architecture of a solid extracellular matrix (ECM) tumor microenvironment could be a key experimental factor in recapitulating DLBCL biology ex vivo. Therefore, we set out to examine the metabolic features of OxPhos-DLBCLs and BCR-DLBCLs in both suspension and Matrigel-based ECM growth conditions.

## RESULTS

### OxPhos-DLBCLs have higher MPC expression and activity than BCR-DLBCLs

**Fig. 1.**
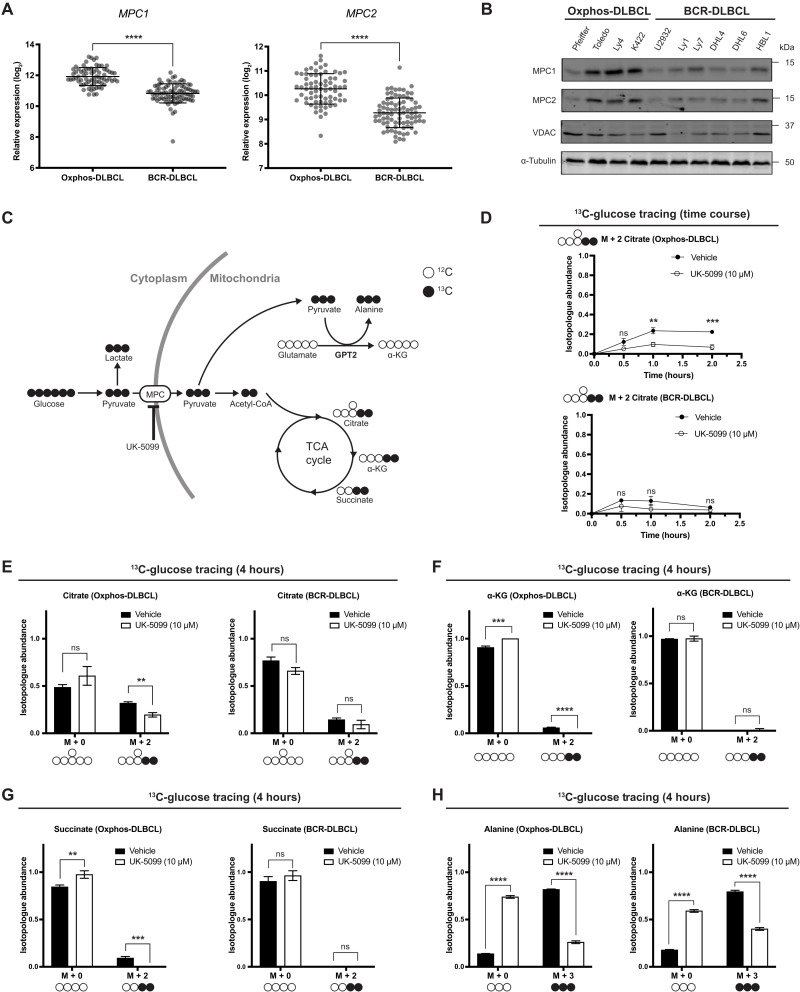
OxPhos- and BCR-DLBCLs have similar pyruvate metabolic profiles. (**A**) Aggregated MPC1 and MPC2 mRNA expression data from 71 OxPhos- and 83 BCR-DLBCL patient samples. Data from GSE10846. (**B**) Western blot analysis of MPC1, MPC2, VDAC, and α-tubulin from a panel of OxPhos- and BCR-DLBCL cell lines. (**C**) Schematic of d-[U-^13^C]-glucose tracing. MPC, mitochondrial pyruvate carrier; UK-5099, MPC inhibitor; α-KG, α-ketoglutarate; GPT2, glutamate pyruvate transaminase 2. (**D**) Quantification of the isotopologue abundance of M + 2 citrate in OxPhos- and BCR-DLBCL cells cultured with d-[U-^13^C]-glucose ± the MPC inhibitor UK-5099 for 30 min, 1 hour, and 2 hours. Isotopologue abundance is the mean of *n* = 3 independent biological experiments, ± SD. (**E** to **G**) Quantification of the isotopologue abundances of M + 0 and M + 2 citrate, M + 0 and M + 2 α-KG, and M + 0 and M + 2 succinate in OxPhos- and BCR-DLBCL cells cultured with d-[U-^13^C]-glucose ± the MPC inhibitor UK-5099 for 4 hours. Isotopologue abundance is the mean of *n* = 3 independent biological experiments, ± SD. (**H**) Quantification of the isotopologue abundances of M + 0 and M + 3 alanine in OxPhos- and BCR-DLBCL cells cultured with d-[U-^13^C]-glucose ± the MPC inhibitor UK-5099 for 4 hours. Isotopologue abundance is the mean of *n* = 3 independent biological experiments, ± SD. Vehicle: Dimethyl sulfoxide (DMSO), ns, *P* > 0.05; *P < 0.05; **P < 0.01; ***P < 0.001; ****P < 0.0001. Data were analyzed by one-way ANOVA followed by Dunnett’s multiple comparison test.

OxPhos-DLBCLs exhibit elevated expression of numerous genes encoding components of the mitochondrial electron transport chain (ETC) compared with BCR-DLBCLs (*12*). Since MPC expression typically correlates with an oxidative metabolic phenotype, we hypothesized that OxPhos-DLBCLs would have higher levels of the MPC than BCR-DLBCLs. Using patient tumor microarray data (GSE10846), we found that the mRNA levels of *MPC1* and *MPC2*, the genes that encode the MPC1 and MPC2 subunits of the MPC, were higher in OxPhos-DLBCLs (Fig. 1A). Furthermore, across 10 DLBCL cell lines, we found that OxPhos-DLBCLs generally had higher MPC1 and MPC2 protein levels than BCR-DLBCLs (Fig. 1B). Analysis of proteomics from isolated mitochondria (*14*) showed that MPC2 was sevenfold more abundant in OxPhos-DLBCLs than BCR-DLBCLs (MPC1-derived peptides, not detected) (fig. S1, A and B). Because MPC1 and MPC2 form an obligate heterodimer and their protein abundances are typically tightly linked, these data suggest that the MPC complex as a whole is up-regulated in OxPhos-DLBCLs (*3*).

We hypothesized that the increased MPC expression in OxPhos-DLBCLs compared with BCR-DLBCLs would result in greater incorporation of carbons from glucose into the TCA cycle through increased mitochondrial transport and oxidation of pyruvate. To test this, we used d-[U-^13^C]-glucose tracing (Fig. 1C). OxPhos-DLBCL (Pfeiffer) cells reached just 25% incorporation of d-[U-^13^C]-glucose into citrate by 2 hours, and treatment with UK-5099, a well-established MPC inhibitor (*17*), further decreased glucose carbon incorporation into citrate to 10% (Fig. 1D). BCR-DLBCL (U2932) cells reached a maximal incorporation of d-[U-^13^C]-glucose into citrate of only 15%, which was also decreased by MPC inhibition, although this change was not statistically significant (Fig. 1D). Thus, the difference in MPC expression levels between the two subgroups is reflected in their glucose-to-citrate labeling, with greater glucose contribution to citrate in the OxPhos subgroup—consistent with a previous study (*13*). However, the overall contribution of glucose to citrate is minimal in both subgroups.

### DLBCLs use mitochondrial pyruvate for mitochondrial alanine synthesis

We were able to measure detectable, albeit low, levels of glucose-to-citrate labeling in DLBCL cells; however, we found even lower labeling of other TCA cycle intermediates, including α-ketoglutarate (α-KG) and succinate, from d-[U-^13^C]-glucose (Fig. 1, E to G, and fig. S1D). This was especially evident in the BCR-DLBCL cell line, where very little α-KG and succinate labeling occurred even after 4 hours (Fig. 1, F and G). Overall, this suggests that glucose does not make a substantial contribution to the TCA cycle in DLBCLs, regardless of their subtype classification. Moreover, MPC inhibition did not increase labeling of pyruvate or lactate in either OxPhos- or BCR-DLBCL cell lines (fig. S1, C and D). This contrasts with other cell types, where inhibiting mitochondrial pyruvate import leads to increased labeling of intracellular lactate, likely to compensate for the loss of adenosine 5′-triphosphate (ATP) production from mitochondrial pyruvate oxidation (*18*). Together, these data suggest that pyruvate minimally contributes to mitochondrial TCA cycle metabolism and ATP production in DLBCLs.

Given the very limited degree to which carbons from glucose were incorporated into the TCA cycle, even in OxPhos-DLBCLs that exhibit higher MPC expression, we sought to uncover the destination of glucose-derived carbon following their entry into mitochondria as pyruvate. Notably, we found a striking incorporation of d-[U-^13^C]-glucose-derived carbons into alanine. This alanine labeling was dependent on MPC activity, as inhibition with UK-5099 substantially decreased labeling in both OxPhos-DLBCL and BCR-DLBCL cells (Fig. 1H and fig. S1, C and D). Thus, despite differences in MPC abundance and pyruvate oxidation in the TCA cycle, alanine is a major fate of glucose carbon in both OxPhos-DLBCLs and BCR-DLBCLs.

### Glutamine feeds the TCA cycle in an MPC-dependent manner

Alanine can be generated by the amination of pyruvate in either the mitochondria or the cytosol. Since inhibiting the MPC, and thus transport of pyruvate into the mitochondria, significantly decreased the ratio of labeled alanine in DLBCLs, our data support a model wherein alanine synthesis is predominantly mediated by the mitochondrially localized glutamate pyruvate transaminase 2 (GPT2) enzyme, which catalyzes the reversible transamination of pyruvate and glutamate to generate alanine and α-KG (Fig. 1C). Although GPT2-mediated glutamate production has been shown to support cell proliferation in non-small cell lung cancer and breast cancer cell lines (*19*, *20*), it is the reverse reaction that produces α-KG, which is important for cell proliferation in the majority of colorectal (*21*–*23*) and other cancers (*24*–*28*).

**Fig. 2.**
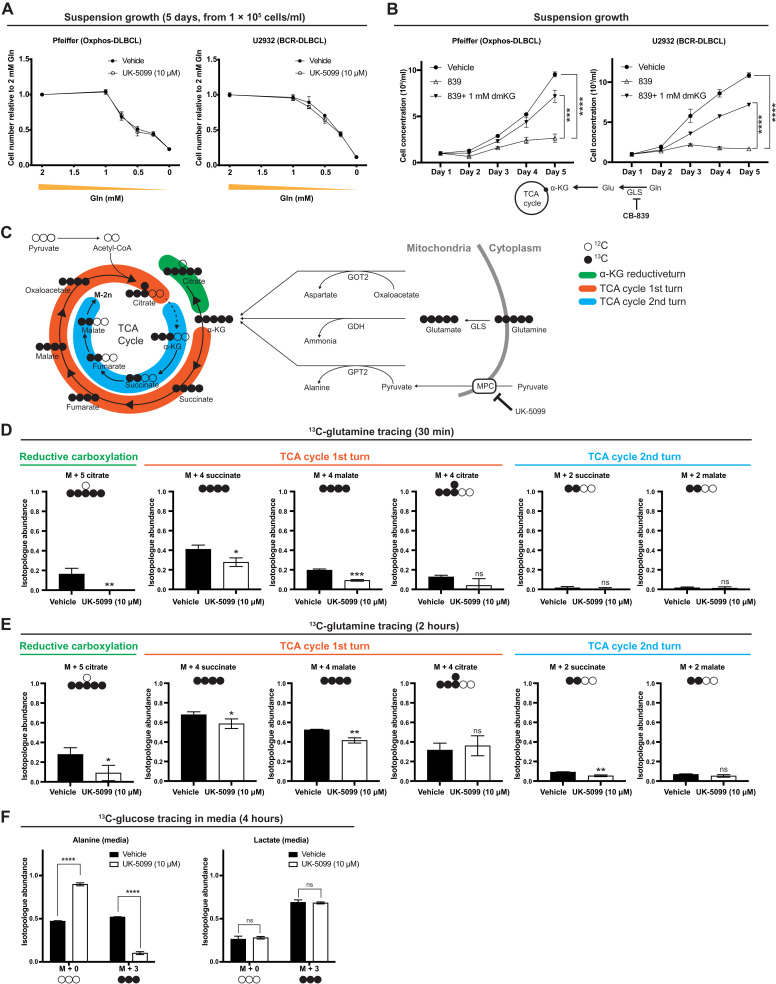
MPC inhibition affects glutamine–to–TCA cycle flux in DLBCL cells. (**A**) Growth assay of OxPhos- and BCR-DLBCL cells cultured in suspension in media supplemented with 2, 1, 0.75, 0.5, 0.25, or 0 mM glutamine ± the MPC inhibitor UK-5099. Cell concentration is the mean of *n* = 3 independent biological experiments, ± SD. (**B**) Growth assay of OxPhos- and BCR-DLBCL cells cultured in suspension and treated with either vehicle, GLS inhibitor CB-839, or CB-839 with dimethyl-α-ketoglutarate (dmKG). Cell concentration is the mean of *n* = 3 independent biological experiments, ± SD. (**C**) Schematic of l-[U-^13^C]-glutamine tracing. GOT2, mitochondrial aspartate aminotransferase; GDH, glutamate dehydrogenase. (**D**) Quantification of the isotopologue abundances of M + 5 citrate, M + 4 succinate, M + 4 malate, M + 4 citrate, M + 2 succinate, and M + 2 malate in DLBCL cells cultured with l-[U-^13^C]-glutamine ± the MPC inhibitor UK-5099 for 30 min. Isotopologue abundance is the mean of *n* = 3 independent biological experiments, ± SD. (**E**) Quantification of the isotopologue abundances of M + 5 citrate, M + 4 succinate, M + 4 malate, M + 4 citrate, M + 2 succinate, and M + 2 malate in DLBCL cells cultured with l-[U-^13^C]-glutamine ± the MPC inhibitor UK-5099 for 2 hours. Isotopologue abundance is the mean of *n* = 3 independent biological experiments, ± SD. (**F**) Quantification of the isotopologue abundances of M + 0 and M + 3 alanine and M + 0 and M + 3 lactate in the medium collected from DLBCLs grown with d-[U-^13^C]-glucose ± the MPC inhibitor UK-5099 for 4 hours. Isotopologue abundance is the mean of *n* = 3 independent biological experiments, ± SD. Vehicle: Dimethyl sulfoxide (DMSO). ns, *P* > 0.05; *P < 0.05; **P < 0.01; ***P < 0.001; ****P < 0.0001. Data were analyzed by one-way ANOVA followed by Dunnett’s multiple comparison test.

Because the GPT2-mediated conversion of glutamate to α-KG requires mitochondrial pyruvate, it is likely dependent on MPC activity. The robust glucose-to-alanine labeling also implies that substantial amounts of glutamine would need to be converted to mitochondrial glutamate for use by GPT2. Therefore, we tested how MPC inhibition affects glutamine consumption by DLBCLs. First, we grew DLBCLs under UK-5099 treatment for 5 days at a series of glutamine concentrations. The proliferation of both OxPhos- and BCR-DLBCL cells was unaffected by UK5099 at all glutamine concentrations (Fig. 2A), suggesting that MPC inhibition does not induce increased glutamine consumption or dependence in DLBCLs. This contrasts with the metabolic responses of glioma cells, cortical neurons, and prostate cancer cells, where MPC inhibition increased glutamine consumption or dependence (*7*, *29*, *30*).

To directly test whether glutaminolysis is important for DLBCL growth, we inhibited the conversion of glutamine to glutamate using the well-established glutaminase (GLS1) inhibitor CB-839 (*31*). Treatment with CB-839 decreased proliferation in both OxPhos- and BCR-DLBCL cells (Fig. 2B and fig. S2A). Furthermore, adding a cell-permeable form of α-KG, dimethyl–α-KG (dmKG), to DLBCLs rescued the effects of CB-839 (Fig. 2B and fig. S2A), indicating that DLBCLs require α-KG generation through glutaminolysis, regardless of their subgroup classification.

To further understand how MPC inhibition affects TCA cycle metabolism, we performed an l-[U-^13^C]-glutamine isotope tracing experiment in BCR-DLBCL cells. We found that 16% of citrate exists as the M + 5 isotopologue, which was completely eliminated upon MPC inhibition (Fig. 2D). M + 5 citrate is indicative of reductive carboxylation, wherein α-KG is converted to citrate through a backward turn of a portion of the TCA cycle (Fig. 2C, green), and this is thought to enable the production of citrate to fuel acetyl–coenzyme A (CoA) synthesis in the cytosol (*32*, *33*). Since the α-KG–to–citrate conversion is reversible, this result suggests that the enzymes that could mediate citrate oxidation, namely, isocitrate dehydrogenase 2 (IDH2) and aconitase 2 (ACO2), are active in DLBCLs.

In contrast to the minimal labeling of TCA cycle intermediates from d-[U-^13^C]-glucose, we observed substantial labeling of M + 4 succinate, M + 4 fumarate, M + 4 malate, and M + 4 citrate from l-[U-^13^C]-glutamine (Fig. 2D and fig. S2B). All of these intermediates are derived from the first turn of M + 5 α-KG through the TCA cycle in the oxidative direction (Fig. 2C, orange). Furthermore, the labeling of these TCA cycle metabolites from glutamine was significantly decreased by inhibiting the MPC (Fig. 2D and fig. S2B). This contrasts with the increased glutamine anaplerosis observed in other cells upon MPC inhibition (*7*, *29*, *30*). As expected, labeling from l-[U-^13^C]-glutamine of glutamate and various isotopomers of TCA cycle intermediates increased after 2 hours, but the same patterns of labeling remained evident (Fig. 2E). MPC inhibition again decreased this l-[U-^13^C]-glutamine labeling, and the dominant isotopomers were from reductive carboxylation of α-KG or the first round of α-KG oxidative conversion through the TCA cycle (Fig. 2E and fig. S2C). These results suggest that DLBCLs have active glutaminolysis and α-KG oxidation and that MPC activity is required for these metabolic processes by enabling GPT2-mediated α-KG production.

The extensive production of alanine from glucose also suggests that alanine could play an important role in DLBCL biosynthetic processes. To address the fate of this alanine, we cultured OxPhos-DLBCL (Pfeiffer) cells with d-[U-^13^C]-glucose for 4 hours and collected the media for isotope tracing analysis. We found that M + 3 alanine is robustly excreted from the cell and that this is dependent on MPC activity (Fig. 2F and fig. S2D). This result supports the idea that α-KG is likely the important product of GPT2 and alanine is primarily a by-product. We also observed M + 3 lactate in the media, but, unlike M + 3 alanine, MPC inhibition did not affect medium M + 3 lactate abundance (Fig. 2F and fig. S2D), similar to our previous findings for intracellular lactate labeling (fig. S1D).

To summarize the above findings using l-[U-^13^C]-glutamine isotope tracing, DLBCLs have an intact and active TCA cycle, but it is primarily fed by glutamine rather than glucose. Although glucose-to-citrate labeling occurred, the glucose-derived carbon in citrate mostly did not progress through the remainder of the TCA cycle. This is likely because of citrate export to the cytosol to support biosynthesis of fatty acid and cholesterol, as well as acetylation events via acetyl-CoA synthesis (*34*, *35*).

### MPC depletion reduces DLBCL proliferation in ECM and in vivo, but not in suspension environment

**Fig. 3.**
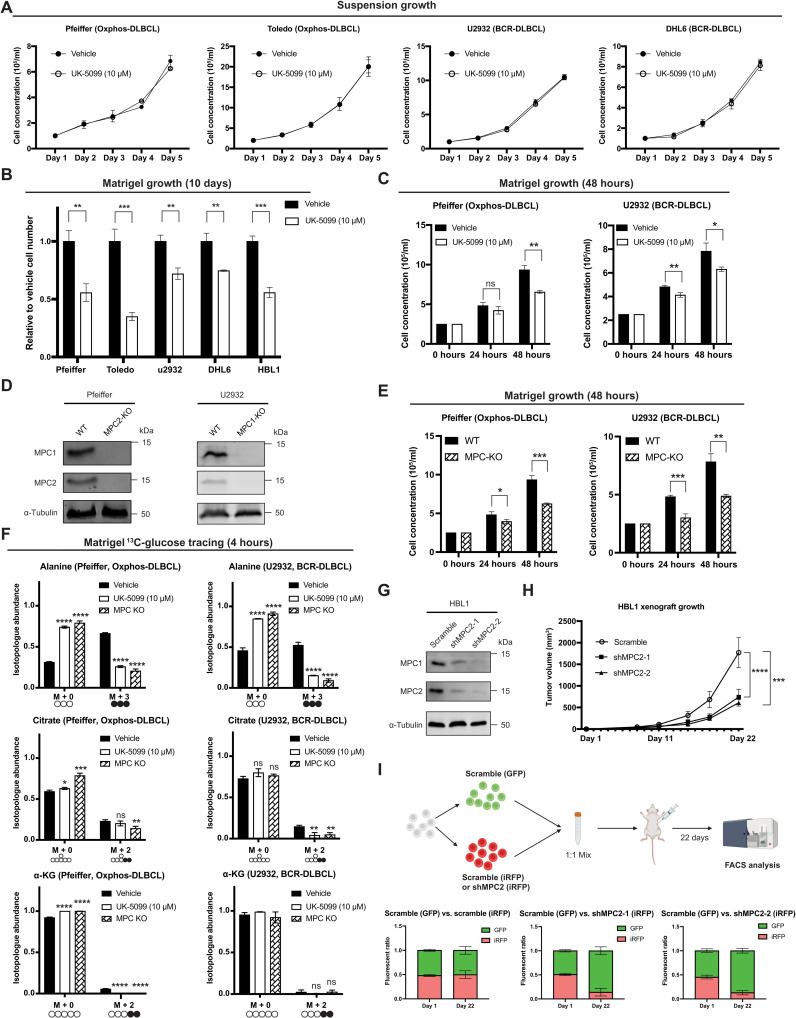
MPC inhibition reduces DLBCL proliferation in Matrigel. (**A**) Growth of cells cultured in suspension ± UK-5099. (**B**) Growth of cells cultured in Matrigel ±UK-5099. (**C**) Growth of cells cultured in Matrigel ± UK-5099. (**D**) Western blot analysis of MPC1, MPC2, and α-tubulin in wild-type (WT) and MPC knockout (MPC KO) DLBCL cell lines. (**E**) Growth of MPC KO cell lines and their WT controls in Matrigel. (**F**) Isotopologue abundances of M + 0 and M + 3 alanine, M + 0 and M + 2 citrate, and M + 0 and M + 2 α-KG from DLBCL WT or MPC KO cells in Matrigel with d-[U-^13^C]-glucose ±UK-5099 for 4 hours. (**G**) Western blot analysis of MPC1, MPC2, and α-tubulin in control (Scramble) and MPC2 knockdown (shMPC2-1 and shMPC2-2) HBL1 cells. (**H**) Xenograft tumor volume of Scramble, shMPC2-1, and shMPC2-2 HBL1 cell lines. Tumor volumes were determined by caliper measurement and are the mean of *n* = 10, ± SEM, analyzed by two-way ANOVA. (**I**) Top: Experiment schematic. Bottom: GFP:iRFP ratios of tumors from Scramble-GFP + Scramble-iRFP, Scramble-GFP + shMPC2-1–iRFP, and Scramble-GFP + shMPC2-2–iRFP in mice at day 1 and day 22. Values are the mean of *n* = 3 (day 1) and *n* = 10 (day 22) ± SD. Vehicle: DMSO. (A to F) Values are the mean of *n* = 3 independent biological experiments, ± SD, analyzed by one-way ANOVA. ns, *P* > 0.05; *P < 0.05; **P < 0.01; ***P < 0.001; ****P < 0.0001.

Given that glutamine is required as a TCA cycle fuel and for DLBCL proliferation, and mitochondrial pyruvate is required to sustain said glutamine oxidation, we hypothesized that loss of MPC function should impair proliferation of DLBCLs. However, inhibiting the MPC in cells grown in suspension culture had no effect on their proliferation (Fig. 3A and fig. S3A). Because DLBCLs form solid tumors (*16*), and we have previously observed that MPC-dependent effects on proliferation were particularly evident in a three-dimensional (3D) environment (*3*), we investigated whether MPC inhibition impairs DLBCL proliferation in Matrigel, an ECM used to mimic the in vivo 3D environment (*36*). Every DLBCL cell line we tested exhibited 30 to 70% fewer cells when cultured with UK-5099 in growth factor–reduced Matrigel for 10 days (Fig. 3B) without any change in viability (fig. S3B). These results indicate that MPC inhibition decreases the proliferation rate of DLBCLs grown in an ECM 3D environment.

When grown in the ECM, DLBCLs form compact colonies within 4 to 5 days of seeding. To address whether this colony formation was necessary for the MPC-dependent decrease in proliferation, we assayed DLBCL cell concentration 24 and 48 hours after plating in ECM. We found that inhibition of the MPC significantly decreased the cell concentration of all four of the DLBCL cell lines that we tested within this time frame (Fig. 3C and fig. S3C). As before, MPC inhibition did not decrease the viability of DLBCLs in ECM at these time points (fig. S3D). We next generated MPC knockout (KO) cell lines from Oxphos-DLBCL (Pfeiffer) and BCR-DLBCL (U2932) cells using CRISPR-based gene disruption (Fig. 3D). As before, MPC depletion had no impact on cell proliferation in the suspension environment (fig. S3E) but significantly decreased their proliferation in ECM by 24 hours (Fig. 3E). These results show that growth in an ECM environment is sufficient to reveal an MPC-dependent growth phenotype in DLBCLs, and this phenotype is not DLBCL subtype specific.

Given the environment-dependent effects on proliferation of MPC inhibition, we asked whether the metabolic effects that we had previously observed in UK-5099–treated suspension cells were also evident in ECM-grown cells. We performed d-[U-^13^C]-glucose tracing experiments with two DBLCL cell lines in ECM. As in suspension culture, alanine labeling was robust and largely MPC dependent—as chemical or genetic ablation of MPC activity significantly decreased the abundance of M + 3 alanine (Fig. 3F). We also observed minimal ^13^C-glucose labeling of TCA cycle intermediates, such as citrate and α-KG, which again was MPC dependent (Fig. 3F). These results confirm that MPC inhibition and genetic ablation have similar effects on glucose metabolism in DLBCLs in both suspension and ECM environments.

Next, we tested whether the proliferation of DLBCL cells is MPC dependent in vivo. Since Pfeiffer and U2932 cells did not form tumors in our xenograft assays, and we were unable to successfully genetically eliminate *MPC1* or *MPC2* in HBL1 cells, we generated HBL1 cell lines wherein *MPC2* was knocked down by stable short hairpin RNA (shRNA) expression (Fig. 3G), and as we have seen previously, depletion of either MPC subunit leads to the loss of the other (*3*, *37*). In xenograft assays, tumors from the *MPC2*-shRNA cell lines grew much more slowly over time compared to the scrambled control (Fig. 3H). To more directly compare engraftment and proliferation of control and *MPC2* knockdown cells, we performed an in vivo competition assay with fluorescently labeled *MPC2*-shRNA cells [infrared fluorescent protein (iRFP)] and Scramble shRNA control cells [green fluorescent protein (GFP) or iRFP]. In this assay, equal numbers of GFP- and iRFP-labeled cells were mixed and injected into mice, and, after 21 days, the tumors were collected and analyzed by flow cytometry to measure the GFP- and iRFP-positive populations. In this assay, both *MPC2*-shRNA cell lines were out-competed by Scramble shRNA control cells (Fig. 3I). Together, these data suggest that MPC is required for efficient DLBCL tumor growth in vivo.

### ECM environment induces DLBCL metabolic reprogramming

**Fig. 4.**
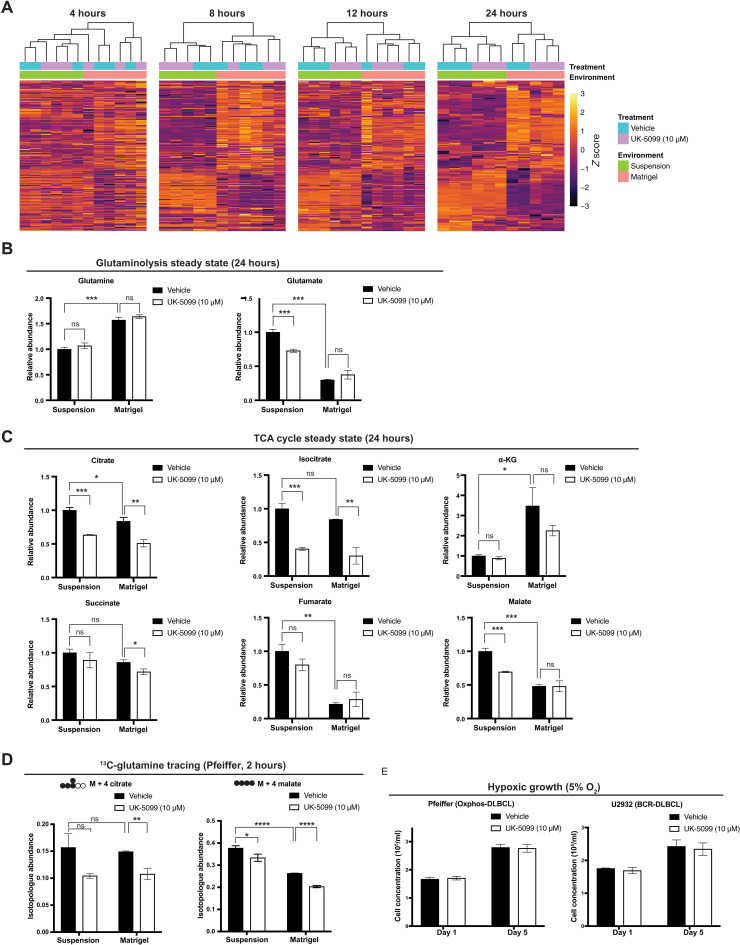
Environmental change reshapes the metabolic landscape of DLBCLs. (**A**) Heatmaps showing the steady-state abundances of metabolites from U2932 BCR-DLBCL cells grown either in suspension or in Matrigel ± the MPC inhibitor UK-5099 for 4, 8, 12, and 24 hours. (**B**) Relative steady-state abundances of glutamine and glutamate from U2932 BCR-DLBCL cells grown either in suspension or in Matrigel ± the MPC inhibitor UK-5099 for 24 hours. Metabolite abundance is the mean of *n* = 3 independent biological experiments, ± SD. (**C**) Relative steady-state abundances of citrate, isocitrate, α-KG, succinate, fumarate, and malate from U2932 BCR-DLBCL cells grown either in suspension or in Matrigel ± the MPC inhibitor UK-5099 for 24 hours. Metabolite abundance is the mean of *n* = 3 independent biological experiments, ± SD. (**D**) Quantification of the isotopologue abundances of M + 4 citrate and M + 4 malate in Pfeiffer DLBCL cells cultured in suspension and in Matrigel with l-[U-^13^C]-glutamine ± the MPC inhibitor UK-5099 for 2 hours. Isotopologue abundance is the mean of *n* = 3 independent biological experiments, ± SD. (**E**) Growth assay of Pfeiffer and U2932 cell lines cultured in suspension ± the MPC inhibitor UK-5099 for 5 days in 5% oxygen hypoxia chamber. Cell concentration is the mean of *n* = 3 independent biological experiments, ± SD. Vehicle: Dimethyl sulfoxide (DMSO). ns, *P* > 0.05; *P < 0.05; **P < 0.01; ***P < 0.001; ****P < 0.0001. Data were analyzed by one-way ANOVA followed by Dunnett’s multiple comparison test.

To understand the full metabolic impact of transitioning from suspension to a solid ECM environment, we collected BCR-DLBCL (U2932) cells for steady-state metabolomic analysis after growth in either suspension or ECM Matrigel, with or without MPC inhibition, for 4, 8, 12, or 24 hours. Through unbiased clustering of both samples and metabolites, we found that 4 hours was sufficient to induce robust changes in the metabolic landscape of ECM-grown cells (Fig. 4A). This change at 4 hours occurred well before we observed a significant impact of MPC inhibition, which is most apparent at the 24-hour time point (Fig. 4A). These results indicate that the growth environment has a broad and relatively rapid impact on DLBCL metabolism. Next, we focused on how this environmental shift affects the glutamine and TCA cycle metabolic phenotypes that we previously observed using isotope tracing experiments. We detected more glutamine and less glutamate in ECM-grown cells than in suspension-grown cells (Fig. 4B). As for TCA-cycle metabolites, α-KG was higher in ECM relative to suspension (Fig. 4C), but MPC inhibition did not affect α-KG abundance in either growth environment (Fig. 4C). We observed decreased abundances of the remaining TCA cycle intermediates in ECM, especially fumarate and malate (Fig. 4C).

Together, these changes result in an increased α-KG:citrate ratio in the ECM environment, which could further increase the reductive carboxylation of α-KG to citrate (*38*). It has also previously been reported that changing from a monolayer culture to spheroid growth enhanced the reductive α-KG to citrate reaction (*39*). Together, these results demonstrate that the ECM environment significantly affects DLBCL TCA cycle metabolism. Furthermore, MPC inhibition consistently decreased citrate and isocitrate abundance in ECM and suspension environments (Fig. 4C). This is likely due to a combinatorial effect of decreased mitochondrial pyruvate to both limit the minimal pyruvate oxidation and to decrease α-KG generation via GPT2. Since the Oxphos- and BCR-DLBCL subgroups both depend on MPC for growth in the ECM environment, it is likely that this metabolic reprogramming also happens in Oxphos-DLBCLs. We also performed an l-[U-^13^C]-glutamine isotope tracing experiment in Pfeiffer OxPhos-DLBCL cells in both suspension and ECM environments. Similar to the BCR-DLBCL (U2932) glutamine tracing experiments performed in a suspension environment, MPC inhibition decreased the fractional labeling of the TCA cycle intermediates citrate and malate from glutamine at 2 hours (Fig. 4D). To test whether hypoxia—which could occur in the ECM environment—underlies the MPC-dependent growth phenotype, we replicated hypoxic growth conditions by culturing DLBCL cells in suspension at 5% O_2_. Hypoxia uniformly decreased the proliferation of DLBCL cells grown in suspension, but MPC inhibition had no further impact on this reduction in growth (Fig. 4E), indicating that the growth phenotype we observe in ECM cannot be explained by hypoxia.

### DLBCLs are sensitive to ammonia in ECM

**Fig. 5.**
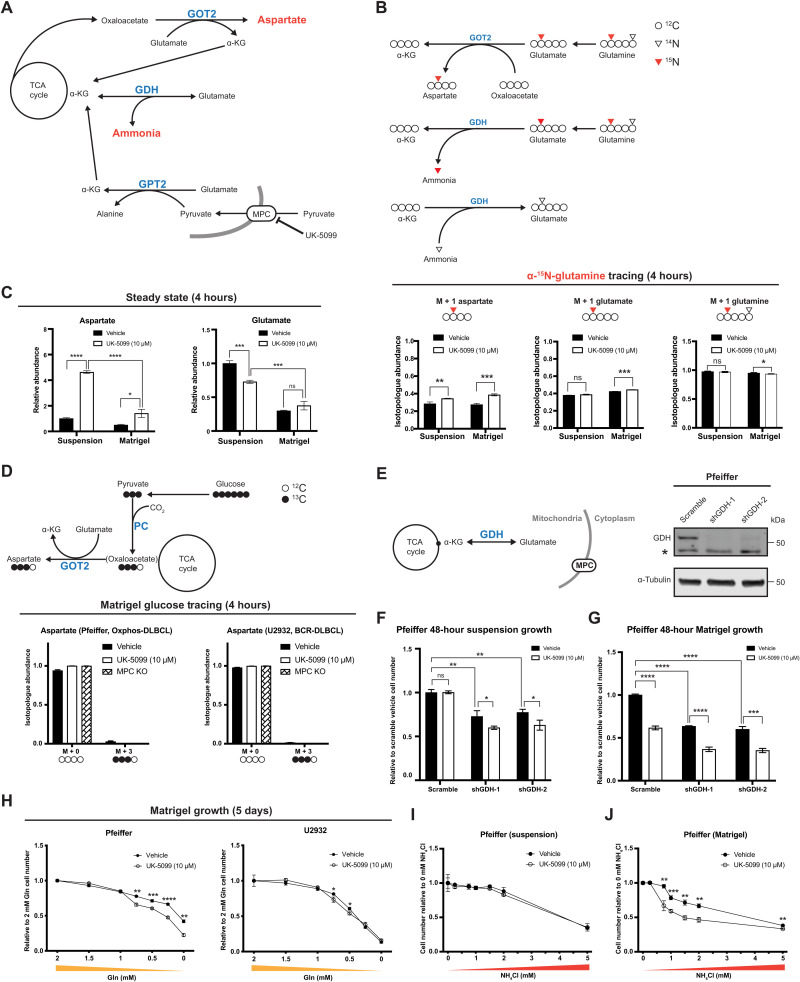
MPC inhibition enhances the sensitivity of DLBCLs to ammonia in Matrigel. (**A**) Schematic of α-KG production by GPT2, GOT2, and GDH. (**B**) Top: Schematic of l-[α-^15^N]-glutamine tracing. Bottom: Isotopologue abundances of M + 1 aspartate, M + 1 glutamate, and M + 1 glutamine in cells cultured in suspension or Matrigel with l-[α-^15^N]-glutamine ± UK-5099 for 4 hours. (**C**) Relative steady-state abundance of unlabeled aspartate and glutamate from U2932 cells cultured in suspension or Matrigel ± UK-5099 for 24 hours. (**D**) Top: Schematic of d-[U-^13^C]-glucose tracing. Bottom: Isotopologue abundances of M + 3 aspartate in cells cultured in Matrigel with d-[U-^13^C]-glucose ± UK-5099 or MPC knockout for 4 hours. (**E**) Left: Schematic of GDH-mediated α-KG production. Right: Western blot of GDH and α-tubulin in Scramble, shGDH-1, and shGDH-2 Pfeiffer cells. *Nonspecific band. (**F** and **G**) Growth of Scramble, shGDH-1, and shGDH-2 Pfeiffer cells in suspension or Matrigel ± UK-5099 for 48 hours. Cell numbers are relative to Scramble with vehicle. (**H**) Growth of Pfeiffer and U2932 cells in Matrigel supplemented with 0, 0.25, 0.5, 0.75, 1, 1.5, or 2 mM glutamine ± UK-5099. Values are relative to 2 mM glutamine with vehicle. (**I** and **J**) Growth of Pfeiffer cells in suspension or Matrigel with 0, 0.3, 0.75, 1, 1.5, 2, or 5 mM NH_4_Cl ± UK-5099 for 48 hours. Values are relative to 0 mM NH_4_Cl ± UK-5099. Vehicle: DMSO values for (B) to (D) and (F) to (J) are the mean of *n* = 3 independent biological experiments, ± SD. *P* value and data analysis are the same as in Fig. 4.

Growth in a solid ECM environment increased α-KG abundance (Fig. 4C); thus, we next asked whether any of the following major α-KG–producing mitochondrial enzymes are responsible for this increase. One candidate is glutamate dehydrogenase (GDH), which converts glutamate to α-KG and produces free ammonia in the process (Fig. 5A). Therefore, excessive GDH activity could be toxic if free ammonia cannot be efficiently cleared (*40*–*42*). Furthermore, it has been reported that GDH can synthesize glutamate from α-KG and environmental ammonia, to both detoxify and recycle ammonia nitrogen for use in biosynthesis processes (*43*). A second α-KG–producing enzyme is the mitochondrial aspartate aminotransferase (GOT2), which converts glutamate to α-KG through consumption of another TCA cycle intermediate, oxaloacetate, and so does not add net carbons into the TCA cycle. (Fig. 5A). The third α-KG–producing enzyme, GPT2, as mentioned previously, consumes glutamate and pyruvate, yielding α-KG and alanine, and thus, its activity is dependent on mitochondrial pyruvate and likely MPC activity (Fig. 5A). Accordingly, the relative contribution of each of these enzymes—GDH, GOT2, and GPT2—to α-KG production can be differentiated on the basis of their consumption and production of specific metabolites.

To determine whether GOT2 activity is increased in response to MPC inhibition, we cultured DLBCL cells in l-[α-^15^N]-glutamine–containing media for 4 hours and analyzed incorporation of ^15^N into aspartate (Fig. 5B). We found that, in both suspension and ECM environments, MPC inhibition increased labeling of M + 1 aspartate (Fig. 5C, right), suggesting that MPC inhibition increases GOT2 activity in both environments. We found that only 42% of glutamate was labeled from l-[α-^15^N]-glutamine at 4 hours (Fig. 5C, right), but 75% of glutamate was labeled from l-[U-^13^C]-glutamine in a similar time frame (fig. S2C). This is likely due to robust ^14^N-glutamate synthesis by GDH from α-KG and environmental ^14^N-ammonia, which has been reported to occur in human breast cancer cells (*43*). Therefore, we hypothesized that this change in GOT2 activity is due to increased cellular demand for α-KG, to compensate for impaired GPT2-mediated α-KG production. In addition, this impaired α-KG production could impair GDH-mediated incorporation of free ammonia into glutamate.

Since glutamine-to-aspartate nitrogen labeling increased upon MPC inhibition, we next questioned whether steady-state aspartate abundance is affected by MPC inhibition. We found that in a suspension environment, MPC inhibition increased aspartate abundance by 4.5-fold, while in an ECM environment, aspartate abundance was only increased by 2-fold (Fig. 5C, left). Because aspartate synthesis is directly tied to GOT2-mediated α-KG production, this result suggests that GOT2-mediated α-KG production might be lower in ECM, perhaps due to decreased availability of oxaloacetate. Therefore, we asked whether glutamate synthesis via GDH is also affected in ECM. We found that the ECM environment caused glutamate abundance to decrease by 70% (Fig. 5C, left). MPC inhibition decreased glutamate abundance by about 30% in the suspension environment, but glutamate abundance was not affected by MPC inhibition in ECM (Fig. 5C, left). This decreased glutamate abundance from suspension to ECM raises the possibility that either the GDH-mediated α-KG and ammonia production could have increased or GDH-mediated ammonia recycling ability could have decreased. As a consequence of this, not enough ammonia can be recycled to glutamate by GDH, resulting in an additional defect in the ability of the cell to detoxify excess ammonia.

To test this model, we first examined the contribution of pyruvate to the TCA cycle through pyruvate carboxylase (PC), which catalyzes the carboxylation of pyruvate to oxaloacetate and thus supports GOT2-mediated α-KG production (Fig. 5D, top). We saw minimal labeling from d-[U-^13^C]-glucose to aspartate in either ECM (Fig. 5D, bottom) or suspension environments (fig. S1D), suggesting that PC and GOT2 are not major contributors of net carbons to the TCA cycle.

Next, we generated Pfeiffer cell lines wherein the gene encoding *GDH* was knocked down by stable shRNA expression (Fig. 5E). We then tested whether these cells are more sensitive to MPC inhibition, as GDH produces α-KG but does not require pyruvate; therefore, loss of GDH might make cells more reliant on GPT2-mediated α-KG production. We expected that *GDH* knockdown not only would have MPC-independent effects on DLBCL proliferation but also would sensitize cells to MPC inhibition in suspension and ECM environments. We observed that *GDH* knockdown decreased DLBCL proliferation in both suspension (Fig. 5F) and ECM environments (Fig. 5G). MPC inhibition further suppressed proliferation of *GDH* knockdown cells in both environments (Fig. 5, F and G), suggesting that GDH is important for the proliferation of DLBCLs in suspension and ECM and is likely acting through a pathway distinct from the MPC and mitochondrial pyruvate.

To further test our model, we asked whether limiting glutamine in combination with inhibiting the MPC could have synergetic effects on cell growth in an ECM environment, even though such synergy was not apparent in a suspension environment (Fig. 2A). We grew DLBCLs in ECM with UK-5099 for 5 days at a series of glutamine concentrations (0, 0.25, 0.5, 0.75, 1, 1.5, and 2 mM). The proliferation of Pfeiffer cells was decreased by UK5099 when glutamine concentration is low (0.75, 0.5, 0.25, and 0 mM), and U2932 cells were also decreased by UK5099 when glutamine concentration decreased to 0.75 and 0.5 mM (Fig. 5H). These results suggest the possibility that GDH activity might be limited in the ECM environment compared to a suspension environment. Next, we tested whether wild-type DLBCLs are more sensitive to ammonia when cultured in an ECM versus in a suspension environment. In suspension conditions, we observed dose-dependent ammonia toxicity with MPC inhibition having no additional effect (Fig. 5I). Cells cultured in ECM were hypersensitive to NH_4_Cl, which was further exacerbated by MPC inhibition (Fig. 5J). In addition, we also observed dose-dependent ammonia toxicity in control cells grown in suspension with MPC inhibition having no additional effect, while MPC inhibition caused a further sensitization to ammonia toxicity in two different *GDH*-shRNA cell lines (fig. S4A). Therefore, DLBCLs grown in ECM are more sensitive to ammonia than cells grown in suspension, and MPC inhibition further increases ammonia sensitivity in ECM-cultured DLBCLs.

To further define the reductive and oxidative modes of citrate synthesis from GPT2-produced α-KG, we also cultured Pfeiffer cells in l-[U-^13^C]-glutamine media for 30 min and analyzed its M + 5 and M + 4 citrate isotopologue abundances change upon piericidin A–induced ETC inhibition. First, we see a significant decrease of ^13^C-glutamine to M + 4 citrate, suggesting that the oxidative conversion of α-KG through the TCA cycle is limited during ETC dysfunction as expected (fig. S4B, left). We did not see decreased labeling of glutamine to M + 5 citrate upon ETC inhibition (fig. S4B, right). Furthermore, this reductive labeling of M + 5 citrate is increased upon addition of pyruvate to the media and decreased upon treatment with the transaminase inhibitor aminooxyacetate (AOA) (fig. S4B, right), suggesting that extra pyruvate addition to cells with an inactive ETC could support the glutamate-pyruvate transaminase reaction.

### BCAA degradation is down-regulated in MPC-inhibited DLBCLs

**Fig. 6.**
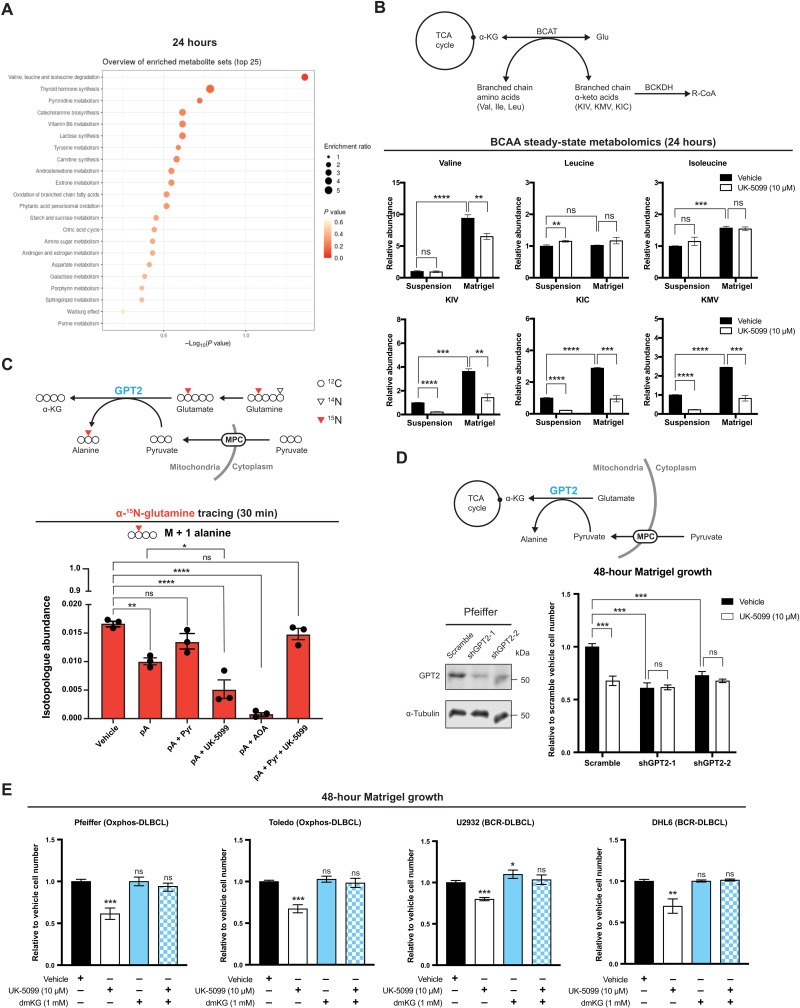
α-KG production supports DLBCL proliferation in Matrigel. (**A**) Metabolite set enrichment analysis of DLBCL cells grown in Matrigel for 24 hours ± UK-5099. (**B**) Top: Schematic of branched-chain amino acid (BCAA) degradation pathway. Bottom: Relative steady-state abundances of metabolites of the BCAA degradation pathway from DLBCL cells grown either in suspension or in Matrigel ± UK-5099 for 24 hours. (**C**) Top: Schematic of l-[α-^15^N]-glutamine tracing with GPT2. Bottom: Isotopologue abundances of M + 1 alanine in Pfeiffer cells cultured in suspension with l-[α-^15^N]-glutamine ± piericidin A (pA; 1 μM), ± sodium pyruvate (Pyr; 1 mM), ± UK-5099 (10 μM), or ± aminooxyacetate (AOA; 500 μM) for 30 min. Piericidin A, ETC complex I inhibitor; UK-5099, MPC inhibitor; AOA, transaminase inhibitor. (**D**) Top: Schematic of GPT2-mediated α-KG production pathway. Bottom left: Western blot analysis of GPT2 and α-tubulin in control (Scramble) and *GPT2* knockdown (shGPT2-1 or shGPT2-2) Pfeiffer cell lines. Bottom right: Growth assay of these cell lines cultured in Matrigel ± UK-5099 for 48 hours. Cell number is relative to Scramble with vehicle treatment. (**E**) Growth assay of DLBCL cell lines cultured in Matrigel and treated with either vehicle, UK-5099, dimethyl–α-KG (dmKG), or UK-5099 with dmKG for 48 hours. Cell number is relative to vehicle treatment. Vehicle: DMSO. Values are the mean of *n* = 3 independent biological experiments, ± SD. ns, *P* > 0.05; *P < 0.05; **P < 0.01; ***P < 0.001; ****P < 0.0001. Data were analyzed by one-way ANOVA followed by Dunnett’s multiple comparison test.

Because MPC inhibition decreased DLBCL proliferation in ECM, we assessed which metabolic pathways were altered under these circumstances. Through a metabolite set enrichment analysis on our steady-state metabolomics data, we found that after 12 or 24 hours of MPC inhibition in ECM, the most affected metabolite sets were those related to branched-chain amino acid (BCAA) degradation pathways (Fig. 6A and fig. S5A). BCAA degradation includes the transamination of BCAAs such as valine, leucine, and isoleucine into their branched-chain keto acids (BCKA), i.e., alpha-ketoisovalerate, ketoisocaproate, and alpha-keto-beta-methylvalerate. In these transamination reactions, α-KG is aminated to glutamate. Therefore, our results suggest that BCAA degradation to BCKAs could be regulated by the MPC through its role in α-KG production. Although the growth environment and MPC inhibition had inconsistent effects on individual BCAAs, BCKAs were all more abundant in ECM than in suspension and decreased upon MPC inhibition (Fig. 6B). These results suggest that MPC inhibition decreases BCKA production presumably by limiting α-KG production.

### GPT2-mediated α-KG production is regulated by the MPC, and controls DLBCL proliferation

To further test whether GPT2-mediated α-KG and alanine production is MPC dependent, we also cultured Pfeiffer cells in l-[α-^15^N]-glutamine–containing media for 30 min and analyzed incorporation of ^15^N into alanine (Fig. 6C, top). We saw that upon piericidin A treatment (to inhibit the ETC), ^15^N labeling into alanine is decreased, and this labeling decrease is diminished upon addition of pyruvate in the media and enhanced upon transaminase inhibitor AOA in the media (Fig. 6C, bottom). These data agreed with our previous data suggesting that GPT reactions could support α-KG production in cells with ETC inhibition (fig. S4B). Furthermore, ^15^N labeling of alanine is further decreased in ETC-inhibited cells upon MPC inhibition (fig. S4B), suggesting that the mitochondrial GPT2 produces α-KG and alanine in an MPC-dependent manner.

To test the hypothesis that the ECM-dependent growth defects caused by MPC inhibition were due to loss of GPT2-mediated α-KG production, we generated a *GPT2* knockdown cell line and a Scramble shRNA control (Fig. 6D). Knockdown of *GPT2* decreased the proliferation of DLBLCs in ECM to a similar extent as MPC inhibition, and, importantly, we found that no additive effect of MPC inhibition (Fig. 6D). These results strongly support our model that MPC inhibition decreases DLBCL proliferation in ECM mainly by restricting mitochondrial pyruvate for the GPT2 reaction.

Last, we added the cell-permeable form of α-KG, dmKG, to cells grown in ECM to determine whether directly increasing α-KG is sufficient to rescue the effects of MPC inhibition. We found that adding dmKG alone had no effect on DLBCL proliferation. However, dmKG completely rescued the MPC inhibition-dependent proliferation defect in all of the cell lines that we tested (Fig. 6E). This further supports the hypothesis that impaired α-KG production is the metabolic defect that underlies the MPC inhibition-induced loss of proliferation in ECM for both Oxphos- and BCR-DLBCLs.

## DISCUSSION

On the basis of the difference in MPC expression between OxPhos- and BCR-DLBCL subgroups, we initially set out to study potential differences in the utilization of mitochondrial pyruvate in these cell types. OxPhos-DLBCLs display a greater incorporation of pyruvate into citrate and are more sensitive to MPC inhibition for this incorporation metric. However, the maximum pyruvate-to-citrate labeling ratio in both OxPhos- and BCR-DLBCL subgroups is low, and labeling of TCA-cycle metabolites downstream of citrate is almost nonexistent. These findings indicate that although there are differences in pyruvate metabolism between OxPhos- and BCR-DLBCLs, these differences are dwarfed by the effects of a common, yet unexpected, source of TCA carbon—pyruvate-enabled glutamine. Nevertheless, in light of a greater understanding of the role of MPC in DLBCLs, it will be interesting to understand the differential expression of the MPC in DLBCL subtypes and whether this leads to additional phenotypes that we have yet to uncover.

**Fig. 7.**
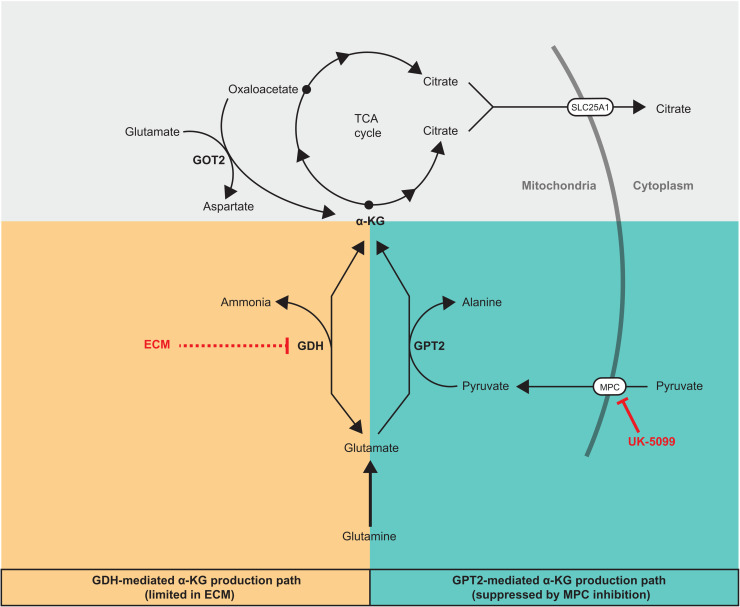
α-KG production pathways that add net carbons to TCA cycle from glutamine.

Although glucose does not substantially contribute to the TCA cycle in DLBCLs, glucose-derived pyruvate facilitates the utilization of glutamine as a TCA cycle fuel by supporting GPT2-mediated α-KG production (Fig. 7). Glutamine tracing experiments demonstrated that glutamine-derived α-KG can be converted to citrate through either the reductive or oxidative modes of the TCA cycle (Fig. 7A). This suggests that DLBCLs have an intact and active TCA cycle, but we consistently observed limited oxidation of citrate to other TCA cycle intermediates. We speculate that this is because citrate is being exported to the cytosol, where it can be used to produce acetyl-CoA for lipogenesis and acetylation, both of which are critical for cancer cells (Fig. 7) (*34*, *35*). In support of this hypothesis, the mitochondrial citrate exporter SLC25A1 appears to be essential in lymphocytes and DLBCLs (fig. S5, A and B). Therefore, DLBCLs appear to have a noncanonical TCA cycle pattern that includes the export of most citrate to the cytosol. This is likely why glutamine, and not glucose, is so prominently incorporated into the TCA cycle in DLBCL cells: Pyruvate-derived citrate is not used to fuel the TCA cycle, but glutamate-derived α-KG is.

We speculate that this previously underappreciated MPC-GPT2–α-KG axis could also play an important metabolic role outside of DLBCLs or other cancers. For example, this metabolic feature might be established during B cell activation. A previous study showed that B cells increase their glucose consumption during activation, but that this increased consumption—reminiscent of our study—does not lead to labeling of TCA cycle metabolites via pyruvate (*44*).

Besides GPT2, GDH is another enzyme that produces α-KG from glutamate and thereby can add net carbons into the TCA cycle (Fig. 7). Because GDH necessarily produces free ammonia while making α-KG, high GDH activity could be toxic to the cell if free ammonia does not diffuse away and cannot be efficiently recycled. Here, we show that transitioning DLBCLs from a suspension environment to a solid Matrigel-based ECM environment makes them more sensitive to ammonia. In addition, MPC inhibition further sensitizes cells to ammonia in this solid environment. We speculate that GDH-produced ammonia does not sufficiently diffuse away from cells in a solid environment, which could feed back on the GDH reaction to prevent α-KG production. As a consequence of this decreased α-KG production, less ammonia can be recycled to glutamate by GDH, resulting in a yet additional defect in the ability of the cell to detoxify excess ammonia (Fig. 7).

A limitation of cell culture has always been an inability to fully recapitulate aspects of an individual cell’s organismal context. For example, the function of pyruvate metabolism for breast cancer metastasis could only be revealed in a solid growth environment (*45*). The importance of this limitation varies depending on cell type. It is now clear that immune cells, including B cells, function in tissues more than in the bloodstream, as previously thought (*46*). Recent studies have reported that the tissue microenvironment could influence DLBCL gene expression (*47*), and physical properties of the ECM environment could also change cancer cells’ mitochondrial structure and function (*48*). Here, we have shown that transitioning DLBCLs from a suspension environment to a solid Matrigel-based ECM rapidly reshapes their metabolome. We found that a growth condition that better recapitulates the in vivo environment is sufficient to unveil entire facets of the DLBCL metabolic landscape not apparent in standard suspension cell culture. Indeed, we identified the environment-specific dependence on MPC activity, which we then recapitulated using in vivo tumor xenograft assays. We anticipate that other aspects of DLBCL biology are also better reflected in an ECM environment, and that the metabolic requirements of many types of solid-tumor cancers may be similarly revealed by more relevant culture systems.

DLBCLs are typically more vascularized compared to follicular lymphoma (*49*, *50*). Aggressive and chemotherapy-resistant DLBCLs often have high vascular endothelial growth factor expression and high microvessel density (*50*–*52*). Besides importing nutrients, tumor blood vessels could also function to export metabolic by-products, such as ammonia and lactate from the tumors. In addition, cancer cells can change their metabolism to adapt to their microenvironment, such that by-products including ammonia and lactate become a nutrient source or anabolic substrate (*43*, *53*). Since MPC inhibition affects DLBCL metabolism and induces ammonia sensitivity, it is possible that a combination therapy of an MPC inhibitor and a drug that blocks tumor blood vessel growth could sensitize cancer cells to their by-product ammonia and also limit essential glutaminolysis.

## MATERIALS AND METHODS

### Experimental model and subject details

#### DLBCL cell lines

DLBCL cell lines used in this study (Pfeiffer, Toledo, OCI-Ly4, Karpas 422, U2932, OCI-Ly1, OCI-Ly7, SU-DHL-4, SU-DHL-6, and HBL-1) have been previously described (*13*, *14*). All DLBCL cell lines were grown in RPMI 1640 medium with glucose (2 g/liter), glutamine (0.3 g/liter; Thermo Fisher Scientific, 11875) supplemented with 10% fetal bovine serum (FBS) (Sigma-Aldrich, F0926), and 1% penicillin/streptomycin (HyClone) at 37°C in a humidified atmosphere containing 5% CO_2_.

#### Stable GDH and GPT2 knockdown cell lines

HEK293T cells were transiently transfected with pLKO.1 shGDH-1 (Sigma-Aldrich, TRCN0000028600), shGDH-2 (Sigma-Aldrich, TRCN0000028611), shGPT2 (Sigma-Aldrich, TRCN0000035028), or Scramble shRNA control (Addgene 8453) along with the lentiviral packaging plasmids pRSV-Rev, pMDLg/pRRE, and pMD2.G using Lipofectamine 2000 transfection reagent. Forty-eight hours after transfection, viral supernatant was collected, filtered through a 0.45-μm polyethersulfone membrane, and stored at 4°C. Polybrene (10 μg/ml; EMD Millipore, TR-1003-G) was added, and a 1:1 mixture of viral supernatant and fresh growth medium (RPMI 1640 + 10% FBS) was applied directly to Pfeiffer cells, which were then incubated for 16 hours at 37°C in a humidified incubator with 5% CO_2_. Viral media were discarded and replaced with fresh growth media, and cells were allowed to recover and expand for 48 hours. After the recovery period, stably infected cells were selected with puromycin (1 μg/ml) for 1 week. Knockdown of GDH and GPT2 was confirmed via immunoblotting as described below.

### Method details

#### SDS–polyacrylamide gel electrophoresis and immunoblotting

Whole-cell lysates (WCLs) were prepared by scraping cells directly into radioimmunoprecipitation assay buffer (50 mM tris-HCl, 1% NP-40, 0.5% sodium deoxycholate, 0.1% SDS, 150 mM NaCl, and 2 mM EDTA) supplemented with protease and phosphatase inhibitors (Sigma-Aldrich, P8340; Roche Molecular, 04906845001), incubated on ice for 45 min with vortexing every 5 min, and then spun at 16,000*g* for 10 min at 4°C to remove insoluble material. WCL was normalized for total protein content via bicinchoninic acid (BCA) protein assay (Thermo Fisher Scientific, 23225). Samples were resolved on SDS–polyacrylamide gel electrophoresis gels and transferred to nitrocellulose membranes. Immunoblotting was performed using the indicated primary antibodies, which are listed in Table 1 according to the manufacturers’ recommendations, and analyzed on a LICOR Odyssey CLx.

**Table 1.** Key resources.

**Reagent or resource**	**Source**	**Identifier**
Antibodies		
MPC1, rabbit monoclonal	Cell Signaling Technology	Catalog no. 14462; RRID:AB_2773729
MPC2, rabbit monoclonal	Cell Signaling Technology	Catalog no. 46141; RRID:AB_2799295
VDAC, rabbit monoclonal	Cell Signaling Technology	Catalog no. 4866; RRID:AB_2272627
GDH, rabbit monoclonal	Cell Signaling Technology	Catalog no. 12793; RRID:AB_2750880
GPT2, rabbit polyclonal	Sigma-Aldrich	Catalog no. HPA051514; RRID:AB_2681516
α-Tubulin, mouse monoclonal	Cell Signaling Technology	Catalog no. 3873; RRID:AB_1904178
		
Bacterial and virus strains		
pLKO.1	Addgene	Catalog no. 8453
		
Biological samples		
		
Chemicals, peptides, and recombinant proteins		
d-[U-^13^C]glucose	Cambridge Isotopes	Catalog no. CLM-1396
l-[U-^13^C]glutamine	Cambridge Isotopes	Catalog no. CLM-1822
l-[α-^15^N]glutamine	Cambridge Isotopes	Catalog no. NLM-1016
Lipofectamine 2000 transfection reagent	Invitrogen	Catalog no. 11668019
Polybrene	EMD Millipore	Catalog no. TR-1003-G
UK-5099	Sigma-Aldrich	Catalog no. PZ0160
CB-839	Sigma-Aldrich	Catalog no. 5337170001
Dimethyl–α-KG	Sigma-Aldrich	Catalog no. 349631
Ammonium chloride (NH_4_Cl)	Sigma-Aldrich	Catalog no. A9434
Matrigel (growth factor reduced)	Corning	Catalog no. 356231
Piericidin A	Cayman Chemical	Catalog no. 15379
Aminooxyacetate (AOA)	Sigma-Aldrich	Catalog no. C13408
Pyruvate	Thermo Fisher Scientific	Catalog no. 11360070
		
Critical commercial assays		
Pierce BCA Assay	Thermo Fisher Scientific	Catalog no. 23225
0.4% trypan blue solution	Sigma-Aldrich	Catalog no. T8154
Deposited data		
Experimental models: Cell lines		
Human: HEK293T cell line	ATCC	Catalog no. CRL-11268; RRID:CVCL_1926
Human: Pfeiffer cell line	Caro *et al.* (*13*)	RRID:CVCL_3326
Human: Toledo cell line	Caro *et al.* (*13*)	RRID:CVCL_3611
Human: OCI-Ly4 cell line	Caro *et al.* (*13*)	RRID:CVCL_8801
Human: Karpas 422 cell line	Caro *et al.* (*13*)	RRID:CVCL_1325
Human: U2932 cell line	Caro *et al.* (*13*)	RRID:CVCL_1896
Human: OCI-Ly1 cell line	Caro *et al.* (*13*)	RRID:CVCL_1879
Human: OCI-Ly7 cell line	Caro *et al.* (*13*)	RRID:CVCL_1881
Human: SU-DHL-4 cell line	Caro *et al.* (*13*)	RRID:CVCL_0539
Human: SU-DHL-6 cell line	Caro *et al.* (*13*)	RRID:CVCL_2206
Human: HBL-1 cell line	Caro *et al.* (*13*)	RRID:CVCL_4213
Recombinant DNA		
pLKO.1 Scramble (Scramble shRNA)	Addgene	Catalog no. 8453
Human shMPC2-1	Sigma-Aldrich	Catalog no. NM_015415; TRCN0000121612
Human shMPC2-2	Sigma-Aldrich	Catalog no. NM_015415; TRCN0000278229
Human shGDH-1	Sigma-Aldrich	Catalog no. NM_005271; TRCN0000028600
Human shGDH-2	Sigma-Aldrich	Catalog no. NM_005271; TRCN0000028611
Human shGPT2-1	Sigma-Aldrich	Catalog no. NM_133443; TRCN0000035028
Human shGPT2-2	Sigma-Aldrich	Catalog no. NM_133443; TRCN0000035024
CRISPR target sequences		
MPC1: AGGTTTACTGGGTTAATTGA and TAGATGCGCTTTAGCAGTTG		
MPC2: AGGGATCGTTGGCAGCCGGG and TGGGTTGGAGTCGTGCGTAA		
Software and algorithms		
Prism 9		
R Project for Statistical Computing	R Core Team, 2020	RRID:SCR_001905
pheatmap	Kolde *et al.* (*55*)	RRID:SCR_016418
ggplot2	Wickham *et al.* (*56*)	RRID:SCR_014601
limma	Ritchie *et al.* (*57*)	RRID:SCR_010943

#### Cell growth and proliferation assays

Cells from suspension culture were removed from flasks, mixed at a 1:1 ratio with 0.4% trypan blue solution (Sigma-Aldrich, T8154), and counted using an automated cell counter (Bio-Rad 1450102). Cells from Matrigel culture were scraped off plates with Matrigel and media, and spun at 600*g* for 10 min at 4°C to remove media and Matrigel. Cell pellets were resuspended in phosphate-buffered saline (PBS), mixed at a 1:1 ratio with 0.4% trypan blue solution (Sigma-Aldrich, T8154), and counted using an automated cell counter (Bio-Rad, 1450102).

#### Matrigel cell culture

Cells were resuspended in ice-cold fresh RPMI 1640 growth medium at 2× concentration and then mixed at a 1:1 ratio with growth factor–reduced Matrigel (Corning 356231). Two hundred microliters of the mix was spotted into seven small drops in one well of a six-well plate. After incubating for 30 min at 37°C and 5% CO_2_ to allow the mix to polymerize, 3 ml of warm RPMI 1640 growth medium was added. Medium was changed every 48 hours.

#### ^13^C-glucose tracing experiments

For suspension-culture tracing, 2 million cells were resuspended in 3 ml of ^13^C-glucose tracing media: glucose-free RPMI 1640 (Thermo Fisher Scientific, 11879020) supplemented with 11.11 mM [U-^13^C]glucose and 10% dialyzed FBS. For Matrigel-culture tracing, 1.5 million cells were plated with Matrigel and then incubated with 3 ml of growth media for 48 hours before the tracing experiment. To start the experiment, media were changed to tracing media. Cells were harvested 30 min, 1 hour, 2 hours, and 4 hours later by centrifuge (scraped then centrifuged for cells from Matrigel) and then quenched with 800 μl of 80:20 methanol:water. Methanol lysates were then subjected to three rapid freeze-thaw cycles and then spun at 16,000*g* for 10 min at 4°C. The supernatants were evaporated using a SpeedVac concentrator.

#### ^13^C-glutamine tracing experiments

For suspension-culture tracing, 2 million cells were resuspended in 3 ml of ^13^C-glutamine tracing media: glutamine-free RPMI 1640 (Thermo Fisher Scientific, 21870076) supplemented with 2.05 mM [U-^13^C]glutamine and 10% dialyzed FBS. For Matrigel-culture tracing, 1.5 million cells were plated with Matrigel and then incubated with 3 ml of growth media 48 hours before the tracing experiment. To start the experiment, media were changed to tracing media. Cells were harvested 30 min and 2 hours later by centrifuge (scraped then centrifuged for cells from Matrigel) and then quenched with 800 μl of 80:20 methanol:water. Methanol lysates were then subjected to three rapid freeze-thaw cycles and then spun at 16,000*g* for 10 min at 4°C. The supernatants were evaporated using a SpeedVac concentrator.

#### ^15^N-glutamine tracing experiments

For suspension-culture tracing, 2 million cells were resuspended in 3 ml of [α-^15^N]glutamine tracing media: glutamine-free RPMI 1640 (Thermo Fisher Scientific, 21870076) supplemented with 2.05 mM [α-^15^N]glutamine and 10% dialyzed FBS. For Matrigel-culture tracing, 1.5 million cells were plated with Matrigel and then incubated with 3 ml of growth media 48 hours before the tracing experiment. To start the experiment, media were changed to tracing media. Cells were harvested 4 hours later by centrifuge (scraped then centrifuged for cells from Matrigel) and then quenched with 800 μl of 80:20 methanol:water. Methanol lysates were then subjected to three rapid freeze-thaw cycles and then spun at 16,000g for 10 min at 4°C. The supernatants were evaporated using a SpeedVac concentrator.

#### Gas chromatography–mass spectrometry derivatization

The supernatants from tracing experiments were evaporated using a SpeedVac. Dried metabolites were resuspended in 30 μl of anhydrous pyridine with methoxyamine hydrochloride (10 mg/ml) and incubated at room temperature overnight. The following morning, the samples were heated at 70°C for 10 to 15 min and then centrifuged at 16,000*g* for 10 min. The supernatant was transferred to a pre-prepared gas chromatography–mass spectrometry autoinjector vial containing 70 μl of *N*-(*tert*-butyldimethylsilyl)-*N*-methyltrifluoroacetamide (MTBSTFA) derivitization reagent. The samples were incubated at 70°C for 1 hour, after which aliquots of 1 μl were injected for analysis. Samples were analyzed using either an Agilent 6890 or 7890 gas chromotograph coupled to an Agilent 5973N or 5975C Mass Selective Detector, respectively. The observed distributions of mass isotopologues were corrected for natural abundance.

#### Steady-state metabolomics experiments

For Matrigel samples, 2 million U2932 cells were plated with Matrigel and then incubated with 3 ml of fresh growth medium in each plate. In parallel, 2 million same passaged cells were resuspended in 3 ml of fresh growth medium in each flask. Cells were harvested 4, 8, 12, and 24 hours later by centrifuge (scraped then centrifuged for cells from Matrigel) and then quenched with 800 μl of 80:20 methanol:water. Methanol lysates were then subjected to three rapid freeze-thaw cycle and then spun at 16,000*g* for 10 min at 4°C. The supernatants were evaporated using a SpeedVac concentrator.

#### Liquid chromatography–mass spectrometry

The metabolite supernatants were evaporated using a SpeedVac. Dried metabolites were reconstituted in 100 μl of 0.03% formic acid in analytical-grade water, vortexed, and centrifuged to remove insoluble material. The supernatant was collected and subjected to screening metabolomics analysis as described on an AB SCIEX QTRAP 5500 liquid chromatography/triple quadrupole mass spectrometer (Applied Biosystems SCIEX) (*54*). The injection volume was 20 μl. Chromatogram review and peak area integration were performed using MultiQuant (version 2.1, Applied Biosystems SCIEX). The peak area for each detected metabolite was normalized against the total ion count of that sample.

#### Xenografts

Ten million HBL1 Scramble or MPC knockdown cells were subcutaneously injected to the flank of the NOD rag gamma (NRG) mice in 1:1 PBS/Matrigel mix. Tumor sizes were measured at the indicated times by a caliper. Animal experiments were conducted in accordance with The University of Utah Institutional Animal Care and Use Committee.

#### Microarray data analysis

Data were obtained from GSE10846 using the package GEOquery (version 2.60.0). BCR and OxPhos classifications were assigned according to Caro *et al.* (*13*). Differential expression between the two groups was determined using limma (version 3.48.1).

### Quantification and statistical analysis

For tracing and steady-state metabolomics, cell growth assays, and cell viability analysis, statistically significant differences were determined using GraphPad Prism 8. Data were analyzed by one-way analysis of variance (ANOVA) followed by Dunnett’s multiple comparison test. A *P* value less than 0.05 was considered to be statistically significant. For microarray data analysis, statistical analysis was performed in R version 4.0.3.

## Notes

View/request a protocol for this paper from *Bio-protocol*.
