# Supplementary material for: Mitochondrial pyruvate supports lymphoma proliferation by fueling a glutamate pyruvate transaminase 2–dependent glutaminolysis pathway
Source: Sci Adv. 2022 Sep 30;8(39):eabq0117. doi: 10.1126/sciadv.abq0117 (PMC9524954; doi:10.1126/sciadv.abq0117)
Supplement: Supplementary file 1 — Figs. S1 to S6 [file sciadv.abq0117_sm.pdf]

Science Advances
AAAS

Supplementary Materials for

Mitochondrial pyruvate supports lymphoma proliferation by fueling a glutamate pyruvate transaminase 2–dependent glutaminolysis pathway

Peng Wei *et al.*

Corresponding author: Jared Rutter, rutter@biochem.utah.edu

Sci. Adv. **8**, eabq0117 (2022)
DOI: 10.1126/sciadv.abq0117

This PDF file includes:

Figs. S1 to S6

Wei et al., Figure S1

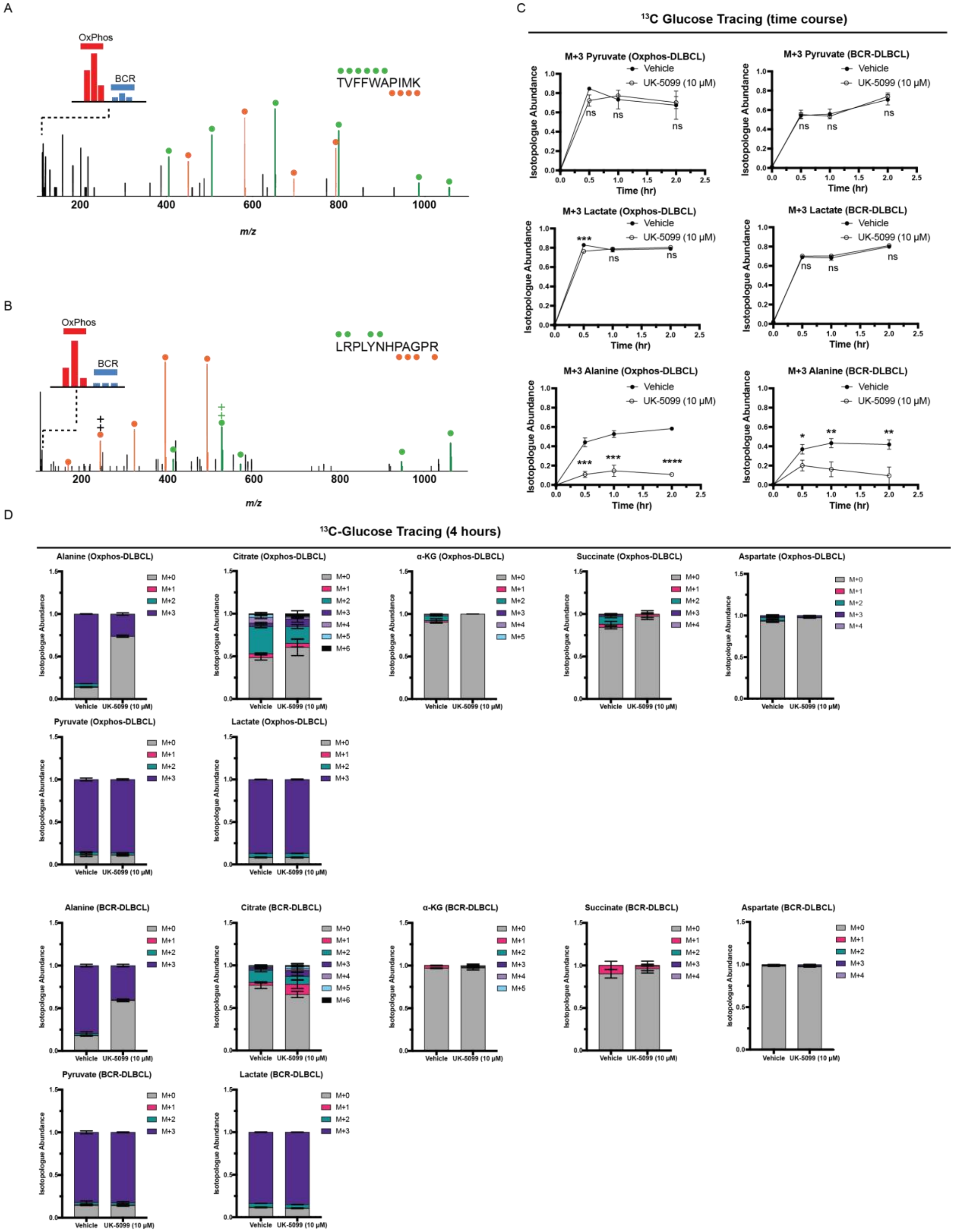

Figure S1. MPC expression and pyruvate metabolism in OxPhos- and BCR-DLBCLs. Related to Figure 1.

(A and B) MS/MS spectra corresponding to MPC2-derived peptides 40TVFFWAPIMK49 (A) and 28LRPLYNHPAGPR39 (B) acquired during multidimensional LC-MS/MS analysis of purified mitochondria from three OxPhos- (Karpas 422, Pfeiffer, and Toledo) and three non-OxPhos/BCR- (Ly1, DHL4, and DHL6) DLBCL cell lines using DEEP SEQ mass spectrometry. Ions of b- and y-type are shown in green and orange, respectively. Relative ratios in BCR- and OxPhos-DLBCL cell lines are derived from iTRAQ reporter ion intensities shown in inset mass spectrum.

(C) Quantification of the isotopologue abundance of M+2 pyruvate, M+3 lactate, and M+3 alanine in OxPhos- and BCR-DLBCL cells cultured with D-[U-^{13}C]-glucose ± the MPC inhibitor UK-5099 for 30 minutes, 1 hour, and 2 hours. Isotopologue abundance is the mean of n = 3 independent biological experiments, ± standard deviation.

(D) Quantification of the isotopologue abundances of alanine, citrate, α-KG, succinate, aspartate, pyruvate, and lactate in OxPhos- and BCR-DLBCL cells cultured with D-[U-^{13}C]-glucose ± the MPC inhibitor UK-5099 for four hours. Isotopologue abundance is the mean of n = 3 independent biological experiments, ± standard deviation.

Vehicle: Dimethyl sulfoxide (DMSO)

ns $p > 0.05$; $^{*}p < 0.05$; $^{**}p < 0.01$; $^{***}p < 0.001$; $^{****}p < 0.0001$. Data were analyzed by one-way Anova followed by Dunnett’s multiple comparison test.

Wei et al., Figure S2

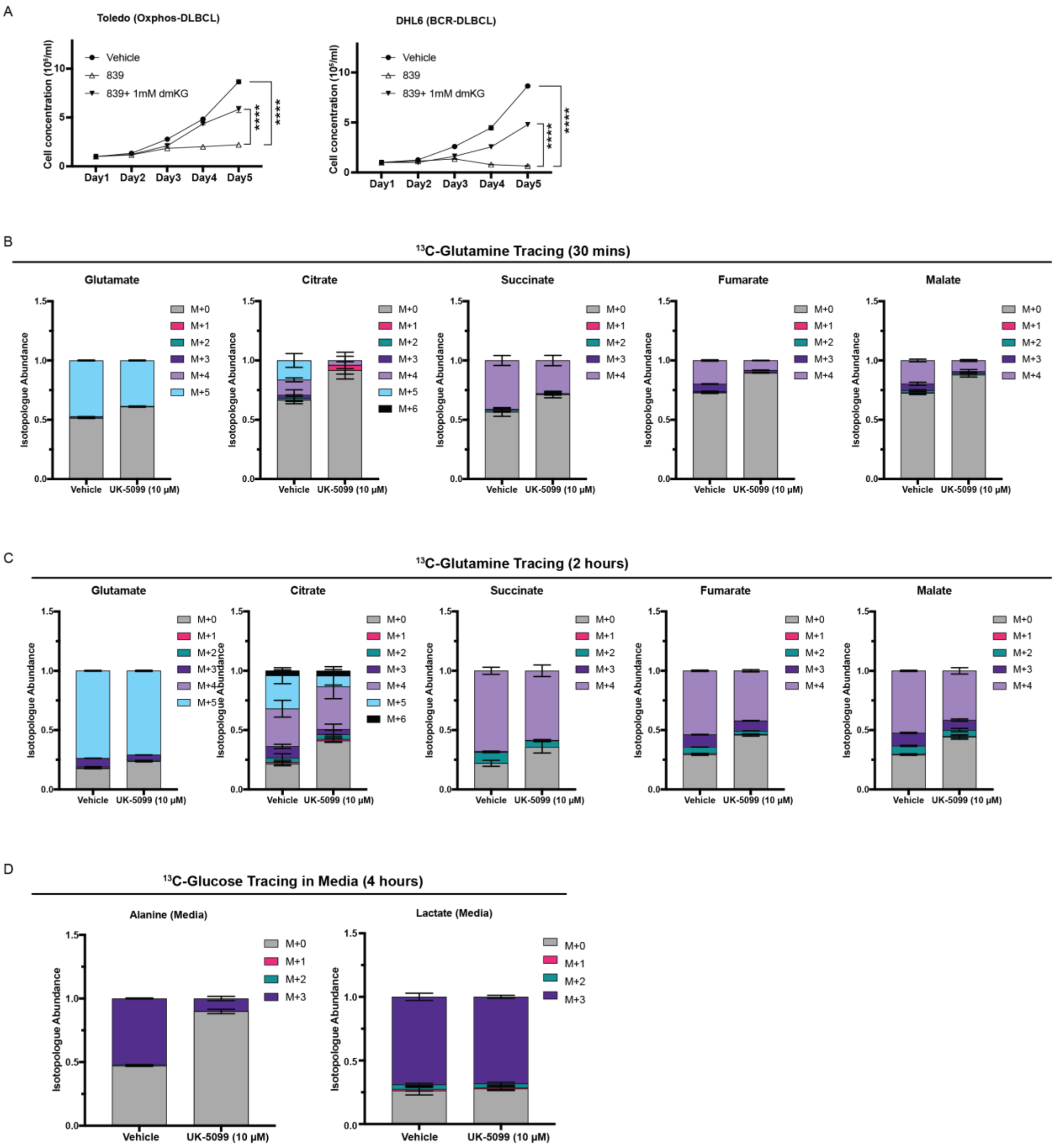

Figure S2. α-KG production is essential for DLBCLs proliferation, and MPC inhibition affects glutamine to TCA cycle flux in DLBCL. Related to Figure 2.

(A) Growth assay of OxPhos- and BCR-DLBCL cells cultured in suspension and treated with either vehicle, GLS inhibitor CB-839, or CB-839 with dimethyl-α-ketoglutarate (dmKG). Cell concentration is the mean of n = 3 independent biological experiments, ± standard deviation.

(B) Quantification of the isotopologue abundances of glutamate, citrate, succinate, fumarate, and malate in DLBCL cells cultured with L-[U-^{13}C]-glutamine ± the MPC inhibitor UK-5099 for 30 minutes. Isotopologue abundance is the mean of n = 3 independent biological experiments, ± standard deviation.

(C) Quantification of the isotopologue abundances of glutamate, citrate, succinate, fumarate, and malate in DLBCL cells cultured with L-[U-^{13}C]-glutamine ± the MPC inhibitor UK-5099 for two hours. Isotopologue abundance is the mean of n = 3 independent biological experiments, ± standard deviation.

(D) Quantification of the isotopologue abundances of alanine and lactate in the medium collected from DLBCLs grown with D-[U-^{13}C]-glucose ± the MPC inhibitor UK-5099 for four hours. Isotopologue abundance is the mean of n = 3 independent biological experiments, ± standard deviation).

Vehicle: Dimethyl sulfoxide (DMSO)

ns $p > 0.05$; *$p < 0.05$; **$p < 0.01$; ***$p < 0.001$; ****$p < 0.0001$. Data were analyzed by one-way Anova followed by Dunnett’s multiple comparison test.

Wei et al., Figure S3

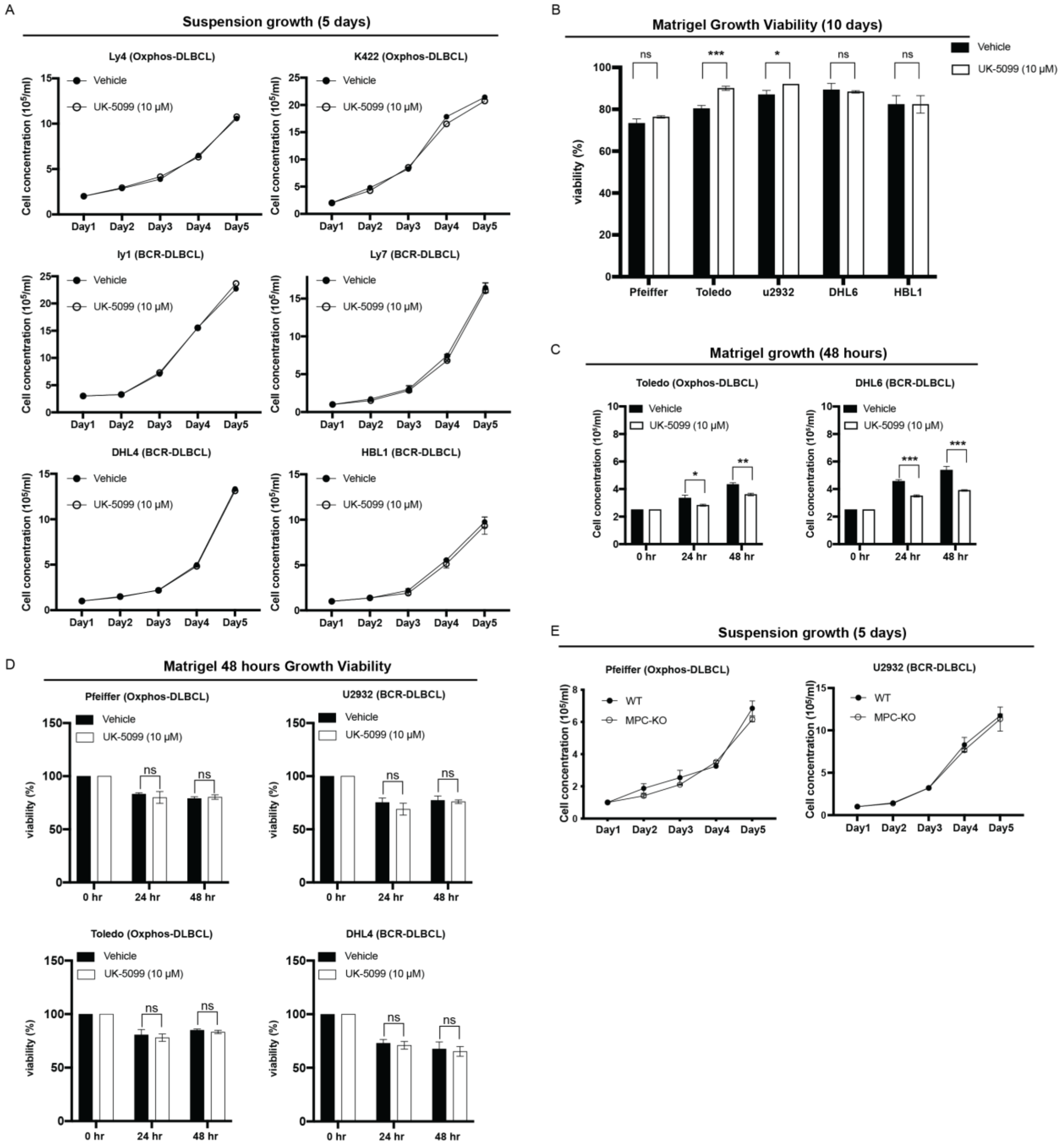

Figure S3. MPC inhibition reduces DLBCL proliferation in Matrigel. Related to Figure 3.

(A) Growth assay of DLBCL cell lines cultured in suspension ± the MPC inhibitor UK-5099 for five days. Cell concentration is the mean of n = 3 independent biological experiments, ± standard deviation.

(B) Cell viability measured by trypan blue staining of DLBCL cell lines cultured in Matrigel ± the MPC inhibitor UK-5099 for 10 days. Cell viability is the mean of n = 3 independent biological experiments, ± standard deviation.

(C) Growth assay of DLBCL cell lines cultured in Matrigel ± the MPC inhibitor UK-5099 for 24 and 48 hours. Cell concentration is the mean of n = 3 independent biological experiments, ± standard deviation.

(D) Cell viability measured by trypan blue staining of DLBCL cell lines cultured in Matrigel ± the MPC inhibitor UK-5099 for 48 hours. Cell viability is the mean of n = 3 independent biological experiments, ± standard deviation.

(E) Growth assay of MPC knock-out (MPC-KO) cell lines cultured in suspension ± the MPC inhibitor UK-5099 for five days. Cell concentration is the mean of n = 3 independent biological experiments, ± standard deviation.

Vehicle: Dimethyl sulfoxide (DMSO)

ns $p > 0.05$; $^{*}p < 0.05$; $^{**}p < 0.01$; $^{***}p < 0.001$; $^{****}p < 0.0001$. Data were analyzed by one-way Anova followed by Dunnett's multiple comparison test.

Wei et al., Figure S4

A

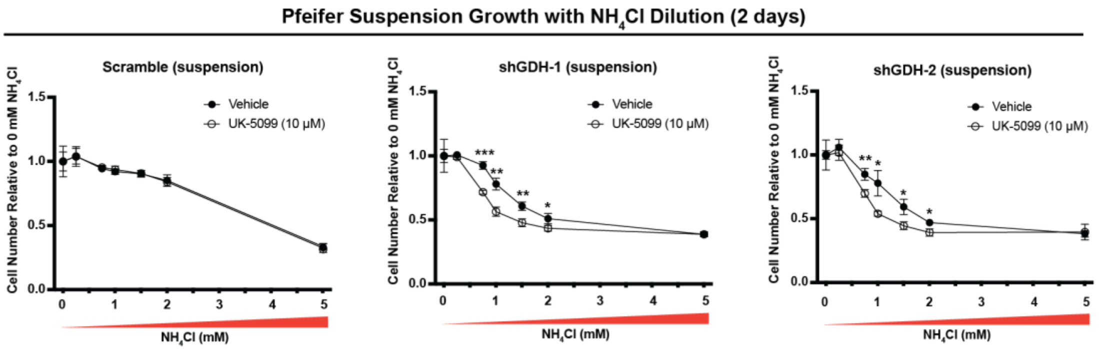

B

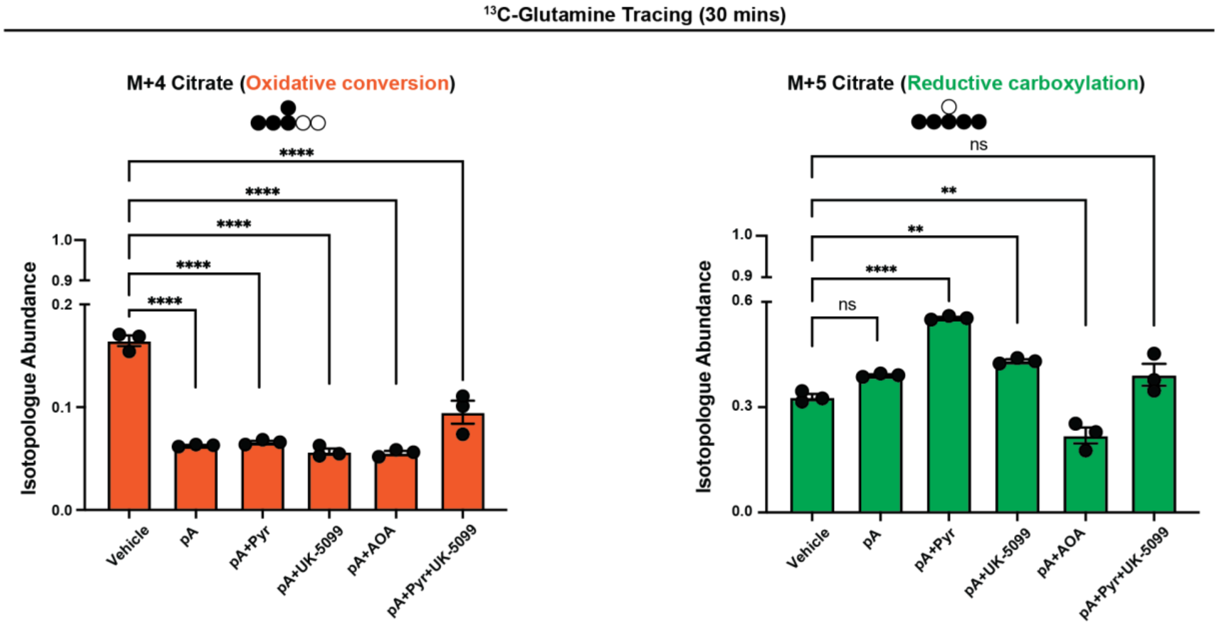

Figure S4. MPC inhibition enhances ammonia sensitivity of GDH knock-down cells; pyruvate supports reductive carboxylation of α-KG to citrate in ETC inactive cells. Related to Figure 5.

(A) Growth assay of control (Scramble) and GDH knock-down (shGDH-1 or shGDH-2) Pfeiffer DLBCL cells cultured in suspension with 0, 0.3, 0.75, 1, 1.5, 2, or 5 mM of NH4Cl ± the MPC inhibitor UK-5099 for 48 hours. Cell number (relative to 0 mM NH4Cl without UK-5099 treatment) is the mean of n = 3 independent biological experiments, ± standard deviation.

(B) Quantification of the isotopologue abundances of M+4 citrate and M+5 citrate in DLBCL Pfeiffer cells cultured with L-[U-^{13}C]-glutamine ± piericidin A (1μM), ± Sodium Pyruvate (1mM), ± UK-5099 (10μM), ± AOA (500μM) for 30 minutes. Piericidin A: ETC complex I inhibitor. UK-5099: the MPC inhibitor. AOA: Aminooxyacetate, the transaminase inhibitor. Isotopologue abundance is the mean of n = 3 independent biological experiments, ± standard deviation.

Vehicle: imethyl sulfoxide (DMSO); pA: piericidin A; Pyr: Sodium pyruvate; AOA: Aminooxyacetate.

ns $p > 0.05$; $^{*}p < 0.05$; $^{**}p < 0.01$; $^{***}p < 0.001$; $^{****}p < 0.0001$. Data were analyzed by one-way Anova followed by Dunnett’s multiple comparison test.

Wei et al., Figure S5

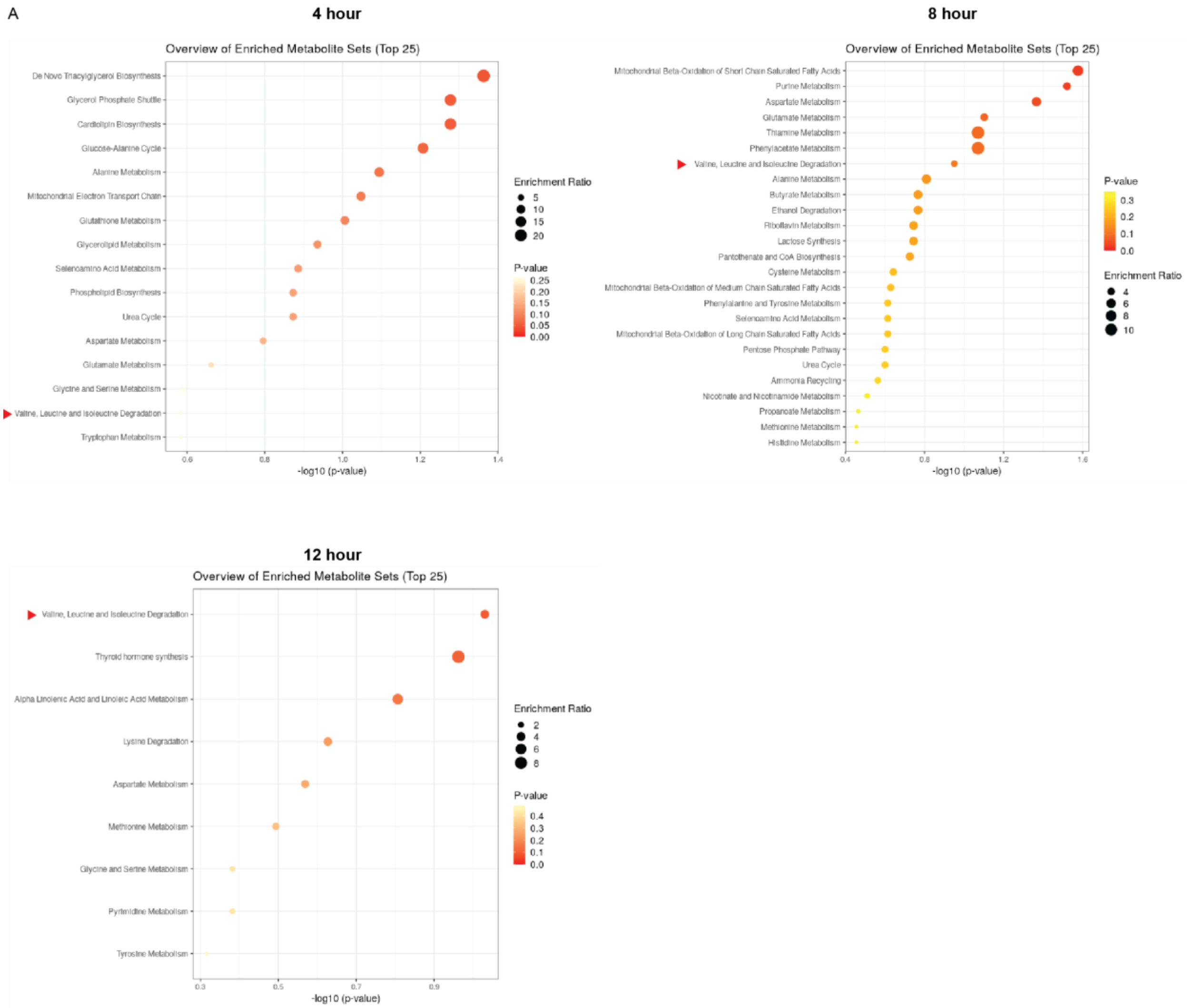

Figure S5. Branched-chain amino acid (BCAA) degradation pathway is affected by MPC inhibition, and α-KG is important for DLBCLs proliferation in Matrigel environment. Related to Figure 6.

(A) Metabolite set enrichment analysis based on MPC inhibition (vehicle vs. UK-5099) of cells grown in Matrigel for 4, 8, and 12 hours. Metabolite abundances are n = 3 independent biological experiments. Arrowheads are pointing to BCAA degradation pathways.

Vehicle: Dimethyl sulfoxide (DMSO)

ns $p > 0.05$; *$p < 0.05$; **$p < 0.01$; ***$p < 0.001$; ****$p < 0.0001$. Data were analyzed by one-way Anova followed by Dunnett's multiple comparison test.

Wei et al., Figure S6

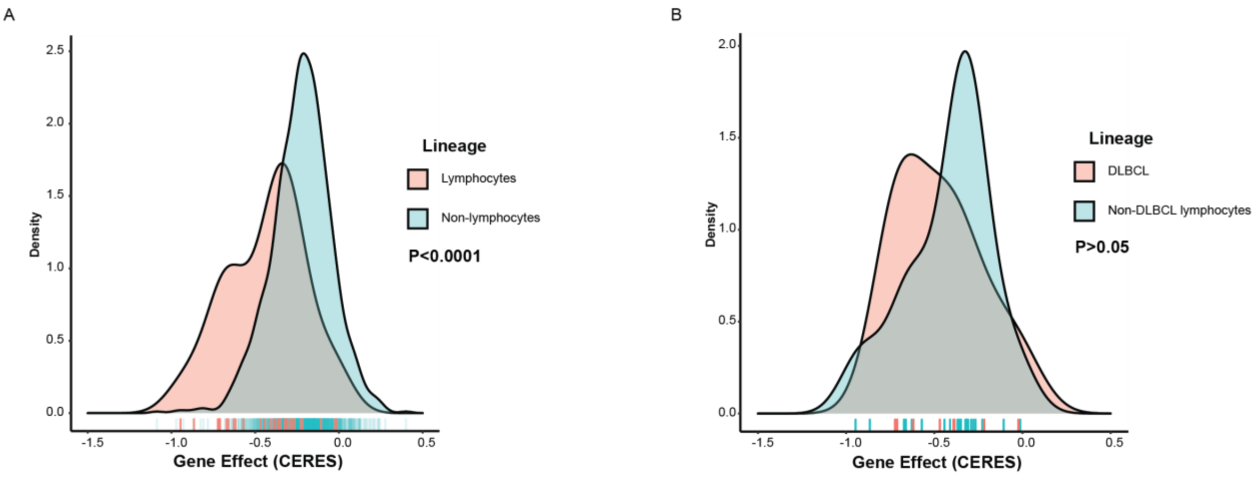

Figure S6. SLC25A1 dependency in cancer cell lines. Related to Figure 7.

(A) Distribution of dependency for SLC25A1 in cancer cell lines of lymphocyte lineage versus cancer cell lines from all other lineages.

(B) Distribution of dependency for SLC25A1 in DLBCL versus other lymphocyte lineage subtypes.

Tick marks indicate individual cell lines. Dependency data downloaded from DepMap release 21Q2.
